# Allosteric Inhibition of Adenylyl Cyclase Type 5 by G-Protein: A Molecular Dynamics Study

**DOI:** 10.3390/biom10091330

**Published:** 2020-09-17

**Authors:** Elisa Frezza, Tina-Méryl Amans, Juliette Martin

**Affiliations:** 1 CiTCoM, CNRS, Université de Paris, F-75006 Paris, France; 2 CNRS, UMR 5086 Molecular Microbiology and Structural Biochemistry, University of Lyon, F-69367 Lyon, France; amans.tina@gmail.com

**Keywords:** molecular dynamics, flexibility, protein-protein interactions, allostery, enzyme activity, docking, homology models

## Abstract

Adenylyl cyclases (ACs) have a crucial role in many signal transduction pathways, in particular in the intricate control of cyclic AMP (cAMP) generation from adenosine triphosphate (ATP). Using homology models developed from existing structural data and docking experiments, we have carried out all-atom, microsecond-scale molecular dynamics simulations on the AC5 isoform of adenylyl cyclase bound to the inhibitory G-protein subunit Gαi in the presence and in the absence of ATP. The results show that Gαi has significant effects on the structure and flexibility of adenylyl cyclase, as observed earlier for the binding of ATP and Gsα. New data on Gαi bound to the C1 domain of AC5 help explain how Gαi inhibits enzyme activity and obtain insight on its regulation. Simulations also suggest a crucial role of ATP in the regulation of the stimulation and inhibition of AC5.

## 1. Introduction

One of the most studied signal transduction pathways is the intricate control of cyclic AMP (cAMP) generation, which is a universal second messenger based on G-protein coupled receptors (GPCR) in eukaryotes [1]. cAMP has a role in a vast number of biological systems, including but not limited to hormone secretion [2], smooth muscle relaxation [3], olfaction [4], learning, and memory [5,6,7].

The family of enzymes responsible for cAMP synthesis are the adenylyl cyclases (also commonly known as adenylate cyclases), which are highly regulated in order to tightly control cAMP levels [8]. Nine mammalian transmembrane ACs are recognized (hereafter termed AC1–9), with a cytoplasmic domain with catalytic properties [8]. Each member of the family has specific regulatory properties and tissue distributions [9,10]; however, they all convert adenosine triphosphate (ATP) into cAMP via a cyclization reaction.

Mammalian ACs share a similar topology of a variable N-terminus (NT) and two repeats of a membrane-spanning domain followed by a cytoplasmic domain [11,12]. The two cytoplasmic domains, called C1 and C2, contain a region of approximately 230 amino acid residues that are roughly 40% identical, which are called C1a and C2a: This implies a pseudosymmetry in ACs. Together, the cytoplasmic domains form the catalytic moiety at the interface. The NT and C-terminal portion of the C1 and C2 domains, called C1b and C2b, are the most variable regions among the different isoforms and can differ among the species. The catalytic site of ACs is located at the C1/C2 interface and binds a molecule of ATP accompanied by two magnesium ions [13].

ACs’ function is regulated by several modulators, which are either stimulators or inhibitors of cAMP synthesis. These include the stimulatory G-protein subunit alpha (Gsα), which is released from its cognate receptor and binds to and activates the AC enzyme via the subunit interaction with the C2 domain [10,14,15,16] upon GPCR activation [14,16,17], the inhibitory G-protein subunits Gαi and Gβγ, calcium ions, calmodulin, and a variety of kinases. AC isoforms integrate several signals and they differ from each other for their modulators and for the different tissues where they are more abundant [18,19,20,21]. Although all nine transmembrane ACs are expressed in the brain, specific ACs are particularly abundant in specific brain regions, and AC5 is highly expressed in the striatum; therefore, it seems to be involved in signal transduction networks that are crucial for synaptic plasticity in the two types of medium spiny neurons [22].

Structural information on the AC cytoplasmic catalytic core [21] and on a complex containing both AC catalytic domains bound to an active conformation of the stimulating Gsα, with or without a bound ATP analog, is available [23]. However, the transmembrane regions contain six predicted membrane-spanning helices each and their function, aside from membrane localization, is unknown. Although the mechanism of stimulation of AC by Gsα is relatively well understood, the mechanism of inhibition of AC activity is still debated: some mutational studies suggest that Gαi binds in an opposite binding site [24], but there are other hypotheses, such as the possibility of the simultaneous binding of Gαi and Gsα or a competition between the two G proteins. However, there are no data on the enzyme bound to ATP (or an ATP analog) in the absence of activating Gsα, on the enzyme in complex with Gαi in the presence or absence of ATP and on the possible trimeric form Gαi + AC + Gsα in the presence or absence of ATP. Hence, it is difficult to understand how Gα subunits activate/inhibit adenylyl cyclase and what is the role of ATP.

To gain insight into the functional mechanism of AC, some studies at the molecular level have been conducted using all-atom molecular dynamics (MD) simulations. In our previous work, we studied the stimulation mechanism of AC5 by performing MD simulations of AC5 alone, AC5 + ATP, AC5 + Gsα, and AC5 + ATP + Gsα [25]. We chose the mouse AC5 isoform among the other isoforms, since this isoform notably plays a key role in a variety of neuronal GPCRs-based signal cascades [19,26,27]. We extensively characterized the flexibility of the four states, the protein–protein interfaces, the ATP mobility, the Gsα binding site, and the Gαi putative binding site on C1 and the effect of ATP and Gsα on these properties. Our study showed that both ATP and Gsα binding have significant effects on the structure and flexibility of adenylyl cyclase. The comparison between the simulations of AC5 + ATP and AC5 + ATP + Gsα helped to explain how Gsα binding enhances enzyme activity and could therefore aid product release. Our simulations also suggested that ATP binding could influence the binding of the inhibitory G-protein subunit Gαi, if the potential binding site within domain C1 were to be involved.

At the same time, another study by Van Keulen and co-workers has been published where they investigated the mechanism of inhibition of AC5 by N-terminal myristoylated Gαi. In their studies, they considered apo AC5 (i.e., without ATP), and they concluded that the myristoylation seems to play a crucial role for the inhibition of AC5. By binding Gαi to a postulated C1 binding site, they found structural modifications that would disfavor both ATP and Gsα binding [28]. Recently, they have also characterized the complex Gαi + AC5 + Gsα using N-terminal myristoylated Gαi [29,30] by comparing the different simulations in order to understand the impact of the binding of both Gα proteins. This comparison suggests that the association of both Gαi and Gsα subunits results in an AC5 conformation similar to that sampled by the Gαi + AC5 complex, indicating that the ternary complex mainly samples an inactive conformation.

**Figure 1:**
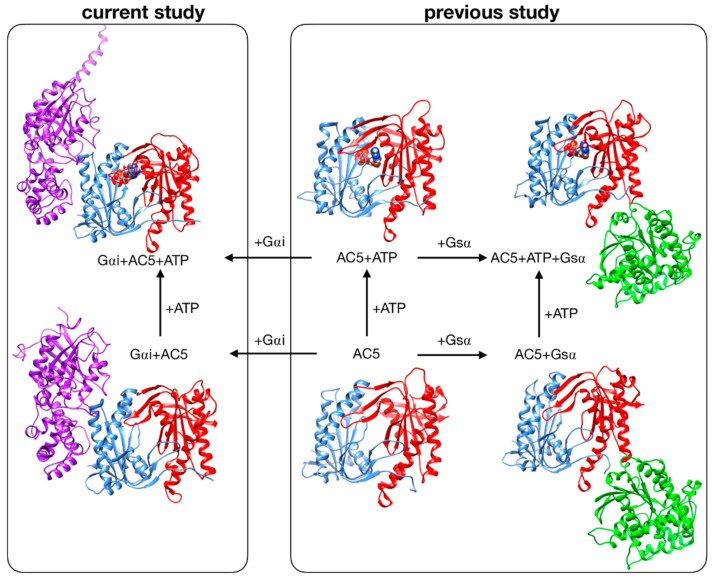
Structure of the cytoplasmic segment of the AC5 isoform of adenylyl cyclase and of its complexes with ATP and the regulating G-proteins Gsα and Gαi viewed from the side closest to the cell membrane. Proteins are shown as backbone ribbons. The C1 and C2 subunits of AC5 are colored blue and red respectively, Gsα is colored green, and Gαi is colored purple. ATP is shown in a CPK representation with standard chemical coloring. In each case, the structures are averages taken from the molecular dynamics simulations. For the AC5 in complex with Gαi with and without ATP, we chose one of the docking poses we used in this work.

Despite these recent studies, the impact that Gαi would have if ATP were already bound in its AC5 pocket and also whether Gsα and Gαi could nevertheless bind simultaneously to AC5 in the presence of ATP are yet to be clarified. In the present study, we used the same approach applied to investigate the stimulation mechanism in our previous work [25]. We have used all-atom molecular dynamics simulations to study the impact of ATP and Gαi on the structure and flexibility of AC5. As in our previous study, we considered only the cytoplasmic domains of AC5, since they are capable of reproducing many of the regulatory properties of the wild-type enzyme and therefore can be used as working models to investigate the regulation mechanisms of AC [31,32]. Since no structural data are available for the complex AC5 + Gαi, we computed docking experiments using representative structures for Gαi and AC5 + ATP obtained from our MD simulations, and we considered two distinctive poses. The all-atom microsecond-scale simulations of AC5 in complex with Gαi with or without ATP studied here (see Figure 1) were compared with our previous simulations of AC5, AC5 + ATP, AC5 + Gsα, and AC5 + ATP + Gsα in order to help explain how binding changes the properties of AC5 and notably to understand the inhibition effect of Gαi.

## 2. Materials and Methods

### 2.1. Models

Models of the cytoplasmic domains of AC5 and Gαi protein were built by homology to known proteins using Modeller v9.12 [33]. In each case, 100 homology models were generated, and the model with the lowest DOPE score [34] was selected. 

For AC5, the model was taken from the MD simulation of our previous study [25] by considering the structure closest to the center of the largest cluster of the last 500 ns simulation of AC5 + ATP. The model we studied earlier [25] was a homology model for the cytoplasmic domains of mouse AC5 (UniprotKB-P84309) with bound ATP, which was generated using the structure 1CJK [13] as template (template/model sequence identity was 98% for the C1 domain, spanning residues 455–643 of P84309, and 57% for the C2 domain, spanning residues 1063–1257 of P84309).

For Gαi, a homology model spanning residues 9–355 of the mouse sequence (UniprotKB-P08752) bound with GSP (a GTP analogue) and Mg^2+^ ion was generated using Modeller with three templates: 1CJK [13] (bovine Gsα 38% sequence identity), 1AS3 [35] (rat Gαi, 81% identity), and 1AGR [36] (rat Gαi, 87% sequence identity). This model was used in docking (see below). In addition, we considered a model of Gαi sampled from MD simulation: a simulation of 1 μs was run starting from the homology model. The structure closest to the center of the largest cluster observed during the simulation was used for docking. In physiological condition, Gαi has post-translational modifications, including a myristoylation on its N-terminus tail that is required for the inhibition of AC5, which affects the conformation and dynamics of Gαi [37]. As the present study is focused on the impact of Gαi binding on the dynamics and conformation of AC5, we did not include such myristoylation in our model of Gαi.

**Figure 2:**
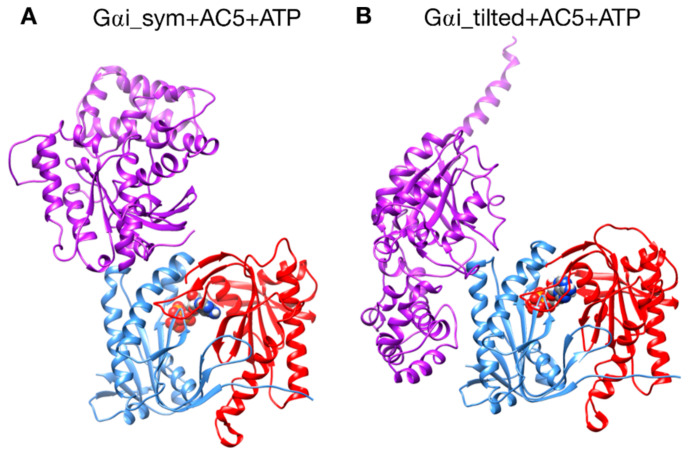
The two different configurations of the Gαi + AC5 + ATP complex simulated in this study. (**A**): Gαi_sym + AC5 + ATP: Gαi has an orientation symmetrical to the Gsα protein in the AC5 + Gsα complex. (**B**): Gαi_tilted + AC5 + ATP: Gαi protein is tilted with respect to AC5.

Models of AC5 and Gαi were docked using CLUSPRO [38,39,40] to generate the Gαi + AC5 + ATP complexes shown in Figure 2, by setting as “attractive residues” (see Figure S1) some residues in the proximity of the presumed binding site from ref [24]. These residues are subject to extra attractive forces during the docking calculation [40].

**Figure 3:**
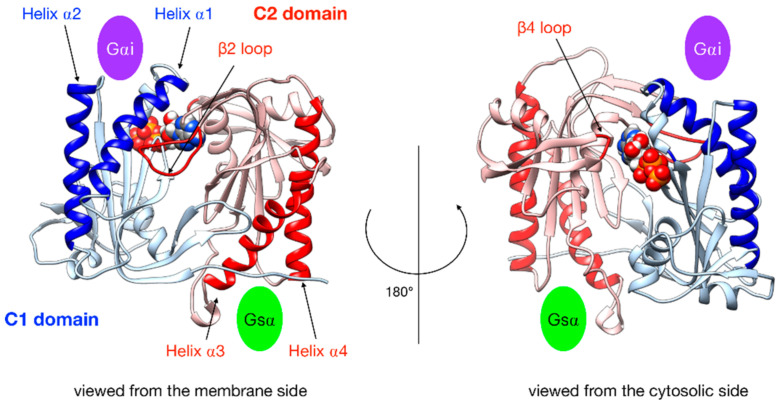
Illustration of the key regions of AC5 catalytic domain structure with bound ATP. The C1 domain is colored blue and the C2 domain is in red, with relevant parts in darker color: the helices of C2 are involved in the binding of the stimulatory protein Gsα, the helices of C1 are involved in the binding of the inhibitory protein Gαi, the β2 loop of C2 (**left side**), and the β4 loop of C2 (**right side**), which bears the catalytic Lysine residue. The green oval indicates the binding site of Gsα, and the purple oval indicates the binding site of Gαi.

A first complex was built by docking the model of AC5 sampled from simulation with the homology model of Gαi. The resulting complex (see Figure 2A) locates Gαi in an orientation similar to Gsα with respect to AC5 in the 1CJK complex, with the Gαi protein binding in the groove formed by the two α-helices termed α1 and α2 in Figure 3. This orientation is called the symmetrical orientation. The resulting complex is denoted Gαi_sym + AC5 + ATP.

A second complex was built by docking the model of AC5 sampled from MD simulation with the model of Gαi sampled from MD simulation. In the resulting complex, Gαi is tilted compared to the Gsα orientation with respect to AC5: the G protein is in contact not only with the helix groove, but also with residues on the side of the C1 domain (see Figure 2B). The resulting complex is denoted Gαi_tilted + AC5 + ATP.

We would like to stress that both Gαi_sym + AC5 + ATP and Gαi_tilted + AC5 + ATP complexes are compatible with the membrane location deduced from the recent full-length structure of AC9 [41], as shown in Figure S2.

Available structures of Gαi display different conformations of the N-terminal helix: either protruding from the structure (in 1AGR) or packed onto the structure core (in 1AS3). In the initial model, this helix was packed. During the simulation of Gαi, this helix appeared very mobile. In order to minimize possible bias and to avoid Periodic Boundary Condition problems in the simulations of Gαi + AC5 complexes, we manually unpacked the N-terminal helix from the structure core after the docking step.

Systems without ATP were also simulated, starting from the same systems after ATP removal. Throughout the study, we compared our results with those obtained in our previous study of AC5 alone and in complex with the activating protein Gsα [25] (see Figure 1, right box).

### 2.2. All-Atom Molecular Dynamics Simulations

Molecular dynamics simulations were performed with the GROMACS 5 package [42,43,44,45,46] using the Amber99SB-ILDN force field for proteins that has been shown to yield an accurate description of many structural and dynamical properties of proteins [47,48,49,50]. Side chain protonation states of titratable amino acids were assigned using a value of pH = 7.4 with the help of the pdb2pqr software [51]. Capping acetyl and methyl-amino groups were added to the N and C termini of both AC5 domains and Gαi to avoid strong artifacts between non-natural termini [23,36]. The four states we study (Gαi_sym + AC5, Gαi_sym + AC5 + ATP, Gαi_tilted + AC5, Gαi_tilted + AC5 + ATP) were each placed in a truncated octahedral box and solvated with TIP3P water molecules [52] to a depth of at least 11 Å. The solute was neutralized with potassium cations, and then K^+^ Cl^−^ ion pairs [53] were added to reach a physiological salt concentration of 0.15 M. Parameters for ATP and GTP were taken from [54]. The parameters for Mg^2+^ came from [55]. This new set of parameters was developed to improve the kinetic properties of Mg^2+^ ions with water and with the phosphate ion, and it was implemented in Amber99. This new set of parameters also provided a better description of the structure of Mg^2+^-phosphate binding than previous sets (these interactions are naturally important in our simulations in the presence of ATP) [55]. Hence, the combination of Amber 99SB-ILDN and the new set of parameters of Mg^2+^ ions is currently the best choice to reproduce the dynamics of AC5 and Gsα, and to properly describe the interactions of Mg^2+^ with AC5 and ATP.

Long-range electrostatic interactions were treated using the particle mesh Ewald method [56,57] with a real-space cutoff of 10 Å. We used virtual interaction sites for the hydrogens, and bond lengths were restrained using P-LINCS [45,58], allowing a time step of 4 fs [59]. The translational movement of the solute was removed every 1000 steps to avoid any kinetic energy build-up [60]. After energy minimization of the solvent and equilibration of the solvated system for 10 ns using a Berendsen thermostat (τ_T_ = 1 ps) and Berendsen pressure coupling (τ_P_ = 4 ps) [61], the simulations were carried out in an NTP ensemble at a temperature of 310 K and a pressure of 1 bar using a Bussi velocity-rescaling thermostat [62] (τ_T_ = 1 ps) and a Parrinello–Rahman barostat (τ_P_ = 1 ps) [63]. Simulations were carried out using typically between 72 and 120 computer cores depending on the system size, which allowed a production rate of about 100 ns/day. Analysis was carried out on a 1.1 μs production segment for each simulation, following a 400 ns equilibration period as in our previous study [25].

### 2.3. Analysis of All-Atom Molecular Dynamics Simulations

We analyzed our all-atom MD simulations using average structures, time-averaged properties such as the RMSD (root mean square deviation), angle between helices, distance between helix axes, distance between the ATP/Mg^2+^ ion and some key residues, and specific geometrical measurements described below, protein–protein and protein–ligand interface characteristics and, in some cases, residue-by-residue conformational and dynamic properties.

When RMSD distributions indicated the existence of distinct conformations, a cluster analysis was carried out using the gromos algorithm of GROMACS [64], using an RMSD cutoff equal to 1.5 Å on backbone atoms, on the conformations collected in the production phase. Clusters accounting for less than 100 ns were discarded.

The C1/C2 interface was characterized using three quantities: the gap volume, the change of accessible surface area upon binding (ΔASA), and the Gap index [65,66]. The Gap index, defined by the gap volume between two protein chains divided by the interface area, measures the shape complementarity at protein–protein interfaces [66]. The gap volume was computed by the SURFNET software [65], and the interface area was calculated using a local implementation of the Lee and Richards algorithm [67] and the same radii.

In order to characterize G protein binding sites, as in our previous work [25], we computed the angle α_C2_ between the pairs of α-helices within domain C2 that bind Gsα (termed α3 and α4 in Figure 3) and also the angle α_C1_ between the quasi-symmetric pair of helices within domain C1 (termed α1 and α2 in Figure 3) that binds Gαi in the present study. The angles were measured using helical axes derived from the residues that remain in stable α-helical conformations throughout the simulations (C1: 408–420 and 468–475, C2: 910–918 and 978–988) as defined in [25]. We also computed the distances between the center of the helices in each domain (d_C1_ and d_C2_, respectively).

To characterize protein–protein interfaces, we computed the interface contacts with the python/C code available at https://github.com/MMSB-MOBI/ccmap, using a fixed cutoff of 5 Å between heavy atoms.

To characterize the ATP binding site, we computed two distances between ATP and two key residues for AC5 activity (distance between O_2_γ and LYS 1065 and between O_2_α and ARG 1029) and the distances between ASP 396 and ASP 440 and the two Mg^2+^ ions [68]. For comparison purposes, we here use the numbering scheme from reference [68]; the corresponding numbering in P84309 is LYS 1245, ARG 1209, ASP 475, and ASP 519.

Data plots were generated with the ggplot2 package in R [69,70], and structure images are generated with UCSF Chimera [71].

## 3. Results

### 3.1. Overview of Simulations

In the absence of any structural information on the catalytic domains of the enzyme with the inhibiting G-protein subunit Gαi, we use a combination of homology modeling, molecular dynamics, and protein–protein docking to get insight on the inhibition mechanism at the molecular level and the impact of the ligand or protein on the conformation and dynamics of AC5.

We have studied the behavior of two molecular species (see Figure 1): AC5 bound to the inhibiting G-protein subunit Gαi (Gαi + AC5) and AC5 bound to both ATP and Gαi (Gαi + AC5 + ATP). For both complexes, we considered two different relative conformations: one called Gαi_sym + AC5 where the Gαi protein has an orientation symmetrical to the Gsα protein in the AC5 + Gsα complex, which is presented in Figure 2A, and one called Gαi_tilted + AC5, where the Gαi protein is tilted, which is presented in Figure 2B. For each of these species, we generated 1.5 μs MD trajectories in an aqueous environment with a physiological salt concentration (0.15 M KCl). The first 400 ns of each trajectory were treated as the equilibration of the system, and analysis was carried out only on the remaining 1.1 μs. In order to understand the effect of Gαi and ATP on AC5, we used the MD simulations for isolated AC5, AC5 with ATP and two Mg2^+^ ions in its active site (AC5 + ATP), AC5 bound to the activating G-protein subunit Gsα (AC5 + Gsα + GTP), and AC5 bound to both ATP and Gsα (AC5 + ATP + Gsα + GTP) obtained in our previous work [25,72]. Data are shown in Tables S1–S5 and in Figures S3–S14.

### 3.2. Stability of Gαi + AC5 Complexes in the Presence and in the Absence of ATP

**Figure 4:**
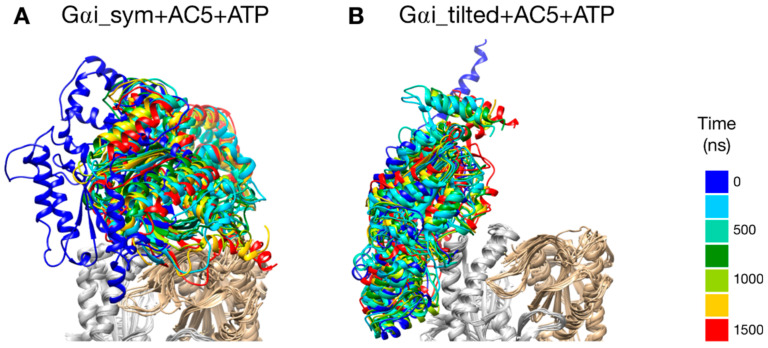
Snapshots of the Gαi + AC5 + ATP complexes observed during the simulations, viewed from the membrane side. Gαi structures extracted every 250 ns are colored on a rainbow scale from blue to red. The C1 domain of AC5 is colored in gray and the C2 domain in beige.

The two types of complexes behave differently in the presence of ATP. In the Gαi_sym + AC5 + ATP simulation, the Gαi protein reallocates significantly with respect to AC5 toward the C2 domain, ending in a configuration where it is in contact with the C2 domain; see Figure 4A. The peculiarity of this system is also apparent in the rest of the study and will be commented on later. On the contrary, in the Gαi_tilted + AC5 + ATP system, the Gαi protein fluctuates around its initial position without significant reallocation, indicating that this complex is very stable; see Figure 4B. We quantified the fraction of interface contacts between Gαi and AC5 that are conserved throughout the simulation time as well as the total number of interface contacts as a proxy of the interface size. For Gαi_sym + AC5 + ATP, the fraction of conserved contacts at the Gαi/AC5 interface drops rapidly below 25%, while the total number of contacts increases significantly (see Figure S3). In Gαi_tilted + AC5 + ATP, 50 to 75% of the initial contacts are conserved during the simulation time, and the total number of contacts also tends to increase, although less dramatically (Figure S3). In the absence of ATP, both systems maintain between 50 and 75% of their initial contacts, with more moderate reallocation and variation in terms of contact number (see Figures S3 and S4).

### 3.3. Impact of Gαi on AC5 + ATP

**Figure 5:**
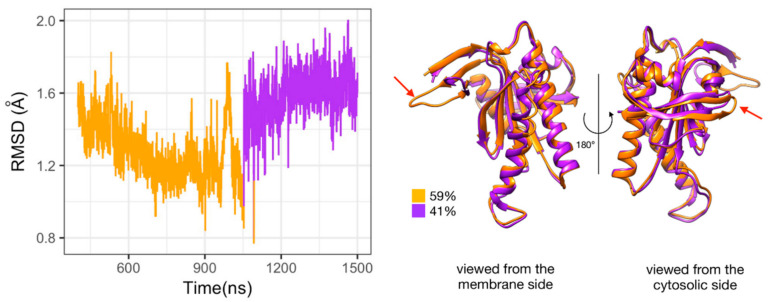
Substates of domain C2 observed during the simulation of Gαi_sym + AC5 + ATP complex. Left side: Root mean square deviation (RMSD) time series for the C2 domain, colored according to cluster membership. Right: structures closest to the center of each cluster, and relative size of each cluster as percentages. Prominent structural changes are indicated by red arrows.

We begin by considering the global impact of Gαi on AC5 + ATP by computing the RMSD on backbone atoms separately on each AC5 domain. RMSD calculations with respect to the average MD structure of each AC5 domain show that Gαi binding has a significant effect on both the structure and the dynamics of the enzyme (see Table S1 and Figure S5 where in order to allow comparison with the results obtained in our previous study [25], values for AC5, AC5 + ATP, AC5 + Gsα, and AC5 + ATP + Gsα were also included). On the one hand, the domain C1 is slightly rigidified by the binding of Gαi. On the other hand, the C2 domain visits several conformational substates involving the ATP binding pocket (β2 loop and β4 loop) in the Gαi_sym + AC5 complex (see Figure 5). These two substates also lead to two different substates for ATP (see Figure S6) which is more mobile, increasing the average RMSD from 0.6 Å for AC5 + ATP to 0.9 Å for Gαi_sym + AC5 + ATP (see Table S1 and Figure S7). In the case of Gαi_tilted + AC5 + ATP, the C2 domain visits a specific substate close to the one sampled in AC5 + ATP where the mobility of ATP is unchanged due to the presence of Gαi.

The presence of several substates upon binding of Gαi is in contrast with the stabilization of a specific substate upon the binding of ATP and/or Gsα. Indeed, in our previous work, we observed that the C2 domain can visit several substates when AC5 is isolated, and the presence of either ATP alone or ATP and Gsα stabilizes two distinct substates. In the former case, a single substate for the β2 loop is selected (the longest-lived substate in isolated AC5) in a closed conformation. In the latter, an opening of loop β2 away from the active site is observed. The selection of a specific substate is correlated to the mobility of ATP and its reactivity [25].

Despite the decrease in flexibility of the C2 domain, which is also observed when Gsα is bound to AC5 + ATP, in the presence of Gαi, ATP is still rather mobile (see Table S1), and for Gαi_sym + AC5 + ATP, an increase in mobility is observed: this impact is opposite to the one observed in AC5 + ATP + Gsα, where a higher stability of ATP is observed (average RMSD equal to 0.3 Å). In both simulations in the presence of ATP, the interactions between the terminal phosphate group of ATP and LYS 1065 (belonging to loop β2) and the interactions between the penultimate phosphate and ARG 1029, a key functional residue, are absent (see Figure S8): the arginine side chain is separated from its target oxygen atom by roughly 9 Å. Moreover, it is known that ATP has stronger interactions with the C1 domain via its associated two Mg^2+^ ions, notably with residues ASP 396 and ASP 440. For Gαi_tilted + AC5 + ATP, these interactions are stable and are not affected by the presence of Gαi. On the contrary, for Gαi_sym + AC5 + ATP, these interactions are absent, justifying the increase of ATP mobility (see Table S3).

**Figure 6:**
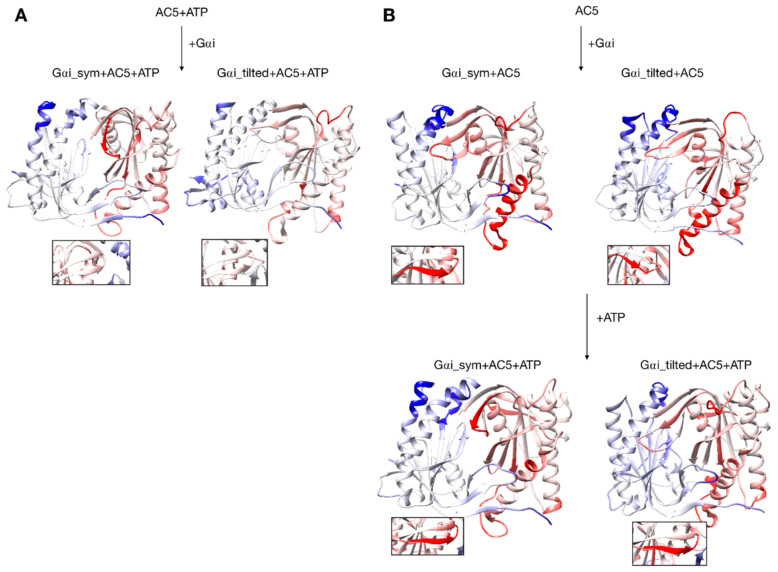
Changes in conformation induced by Gαi protein. (**A**) scenario where ATP is already bound to AC5 when Gαi interacts, (**B**) scenario where ATP is not yet bound to AC5 when Gαi interacts. More intense colors (blue for domain C1 and red for domain C2) correspond to larger movements compared to the preceding structure (i.e., AC5 + ATP for A and AC5 and AC5 + Gαi for B) on a scale of 0 to 4 Å. The insets display the β4 loop.

**Figure 7:**
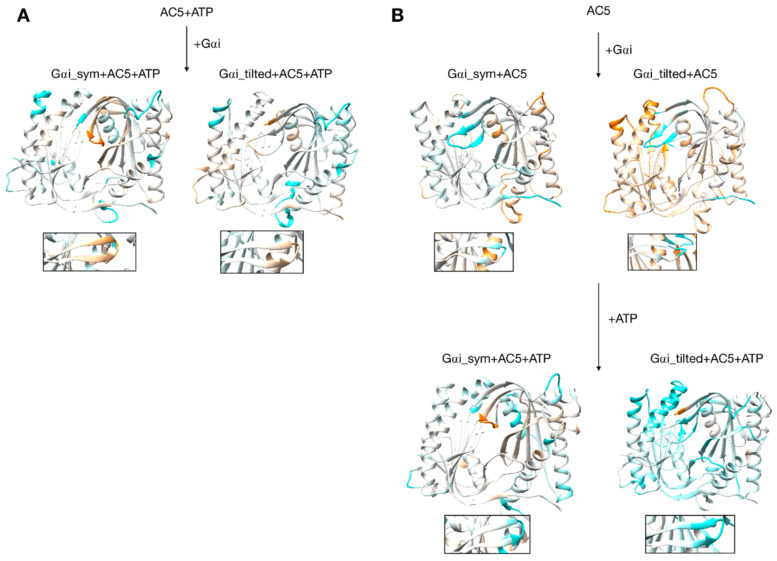
Changes in flexibility induced by G proteins. (**A**) Scenario where ATP is already bound to AC5 when Gαi interacts, (**B**) scenario where ATP is not yet bound to AC5 when Gαi interacts. More intense colors (orange for increased flexibility and cyan for decreased flexibility) correspond to differences with respect to the preceding structure on a scale of −1.2 to +1.2 Å. The insets display the β4 loop.

Gαi binding also turns out to have more global effects on AC5 + ATP. First, the angles and the distance between the pairs of α-helices in both AC5 domains are modified. In Gαi_sym + AC5 + ATP, the angle between the helices α1 and α2 in domain C1 is significantly reduced (by 9°, see Table S1) and the angle between the helices α3 and α4 in domain C2 is slightly increased (by 4°). In Gαi_tilted + AC5 + ATP, an opposite effect is observed: the angle between the helices α1 and α2 in domain C1 is hardly affected (increased by 1°, see Table S1 and Figure S9), and the angle between the helices α3 and α4 in domain C2 is slightly decreased (by 3°). In addition, the distance between the C2 helices in Gαi_tilted + AC5 + ATP complexes is maintained around 13 Å, whereas our earlier results indicate that it is around 16 Å in the AC5 + ATP + Gsα complex (see Table S1 and Figure S10). In both simulations, the C1/C2 interface remains mostly as tight as in isolated AC5 + ATP (gap index from 2.8 Å for AC5 + ATP to 2.9 Å once Gαi is bound, Table S1 and Figure S11) involving a movement of helix α3 (see Figure 6). In terms of flexibility, Gαi binding mainly flexibilizes the binding site region of ATP in AC5, although it also decreases the flexibility of the C-terminals of helices α1 and α3 (see Figure 7A).

### 3.4. Impact of Gαi on Apo AC5 and Further Impact of ATP on AC5 + Gαi

Although it seems probable that ATP is already bound to AC5 based on our previous study [25], we also consider the scenario where ATP is not already present when Gαi binds on AC5. We begin by considering the global impact of Gαi on AC5. Gαi_sym and Gαi_tilted have different effects on the C1 domain of AC5: Gαi_sym slightly rigidifies it as attested by the RMSD calculation (Table S1 and Figure S5), whereas Gαi_tilted flexibilizes it, and this flexibility concerns the binding helices α1 and α2 (Figure 7B). For the C2 domain, in both Gαi_sym + AC5 and Gαi_tilted + AC5 complexes, the β2 loop and the β4 loop are rigidified upon the addition of Gαi (see Figure 7B). Their conformations slightly differ in both complexes: the β2 loop is more closed with Gαi_tilted than with Gαi_sym, whereas the β4 loop, at the back of the structure, is half open with Gαi_tilted compared to with Gαi_sym (see Figure S12). In Gαi_sym + AC5, the C2 domain visits several conformational substates involving the helices α3 and α4 (see Figure S13).

The conformation of the binding helices in both AC5 domains is significantly altered by Gαi. Gαi notably displaces helix α3 (Figure 6B). In both Gαi_sym + AC5 and Gαi_tilted + AC5, the angle between the helices α1 and α2 in domain C1 is significantly increased (by 24° and 19°; see Table S1 and Figure S9). On the contrary, the angle between the helices α3 and α4 in domain C2 is significantly decreased (by 7° and 12°), and the helices are also closer to each other (Table S1 and Figure S10). The C1/C2 interface remains as tight as in isolated AC5, in contrast with what we observed previously with the binding of Gsα, which resulted in a looser C1/C2 interface (gap index equal to 3.8 Å, see Table S2 and Figure S11).

In the scenario where ATP is not already bound to AC5 when Gαi interacts, we can also analyze the effect of ATP addition on the pre-formed Gαi + AC5 complex. As shown in Figure 7B, the addition of ATP notably rigidifies AC5. The interface at the Gαi/AC5 interface is quite loose in the Gαi_tilted + AC5 complex (gap index equal to 4.6 Å, see Table S2), and the addition of ATP tends to tighten this interface (gap index equal to 4.2 Å). On the contrary, in the Gαi_sym + AC5 complex, the initial Gαi/AC5 interface is made more loose by the addition of ATP (gap index from 3.2 Å in Gαi + AC5 to 5.4 Å when ATP is bound), which is probably due to the change of interface as observed by the losing of 75% of the native contacts by adding ATP (see Figure S3). By comparison, in the AC5 + Gsα complex, the gap index decreased from 3.2 to 2.7 Å upon ATP addition (Table S2). Despite the variation of the values of gap index for the Gαi/AC5 interface upon the binding of ATP, all the values are typical of obligate protein–protein interfaces [73].

## 4. Discussion

As already observed in our previous work [25], microsecond-scale simulations are necessary to investigate the allosteric coupling existing within AC5 and the effect of the binding of Gαi. Similarly to van Keulen and Rothlisberger [28], we studied the scenario where Gαi binds to AC5 in the absence of ATP, but we could not exclude the possibility that ATP is bound on AC5 when Gαi binds, based on our previous work [25]. Due to the lack of structural information on the complex between Gαi and AC5, we generated docked models using CLUSPRO, which is a server that achieves good results in the blind community-wide assessment CAPRI [74]. In order to reduce possible structural bias, we used as input isolated protein models sampled from MD simulations and integrated putative interface residues [24] in the form of a soft constraint by specifying attractive residues for the docking calculation. We considered two different docking poses in our study: Gαi_sym and Gαi_tilted. In the former, the Gαi protein is bound to AC5 in a symmetrical fashion compared to what is known for the AC5 + ATP + Gsα complexes (Figure 2A) and similar to the model postulated by Dessauer et al. [24]. In the latter, the Gαi protein is rotated and tilted onto the C1 domain (Figure 2B). Both complexes stay bound during the simulations, but a greater stability is obtained for the Gαi_tilted configuration (see Figure 4), suggesting more biological relevance. In the case where Gαi binds on AC5 + ATP in a symmetrical fashion, an allosteric effect is observed: a closure on the Gαi site is coupled with an opening on the Gsα site and an opening of the β2 loop of C2, as observed for the binding of Gsα. Despite that, this conformation seems to be less likely because the complementarity is very low and the interface is unstable, as already mentioned above. On the contrary, the Gαi_tilted configuration is also very similar to the one reported by van Keulen and Rothlisberger in their recent work where they studied the complex between myristoylated Gαi and AC5 in the absence of ATP [28], although the starting conformations of AC5 and Gαi are quite different, as well as the docking procedures. Given the inherent complexity of protein–protein docking, such convergence toward similar models despite different approaches is an additional support to the biological relevance of this model. 

On the experimental side, several mutations on AC5 are known to impact its regulation by Gαi: ASP411, MET414, THR415, and ASP418 in helix α1, LEU472 and VAL476 in helix α2, and VAL479 in the loop connecting helix α2 to a β-strand [24] (see Table S4 for the numbering scheme). In our simulations of the Gαi_tilted + AC5 complex, these residues are remarkably persistent, i.e., present more than 80% of the time at the interface between AC5 and Gαi with and without ATP; see Table S4. Not only are these residues persistent at the interface, but they also form stable interactions with particular side chains on Gαi belonging to the switch I, switch II and switch III regions [75] (see Table S5 and Figure S14). The high stability of such interactions observed in our MD simulations suggest a functional role for these residues, which is consistent with the fact that the mutations affecting them have an impact on the regulation of AC5 by Gαi, as observed experimentally [24].

The binding of Gαi slightly rigidifies the C2 domain in all simulations, and an opening of the Gαi binding site is coupled with a closure of the Gsα binding site, in particular in the Gαi_tilted + AC5 complexes with and without ATP, as observed in van Keulen and Rothlisberger’s simulation [28]. As in the case of the AC5 + Gsα complex, ATP stabilizes the Gαi/AC5 interface when Gαi is tilted, whereas the latter is less complementary. All these changes involve coupling through AC5 over distances of tens of angstroms.

**Figure 8:**
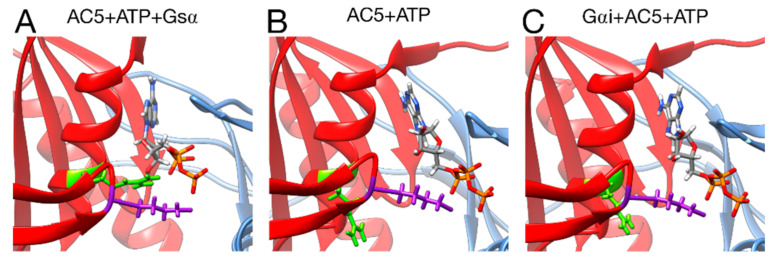
Interactions between ATP and key residues in different complexes. (**A**) active AC5 + ATP + Gsα, (**B**) inactive AC5 + ATP complex, (**C)** inactive Gαi_tilted + AC5 + ATP complex. Key residues of C2 are shown as sticks and colored in purple (LYS 1065) and green (ARG 1029). The C1 and C2 subunits of AC5 are colored blue and red, respectively. For clarity, the region 394–428 of C1 is omitted from the representation.

Turning now to the enzymatic function of AC5, it is known that specific hydrogen bonds between AC5 and ATP play an important role in the production of cAMP from ATP, as already shown in hybrid QM/MM free energy calculations, notably the hydrogen bond between the highly conserved ARG 1029 and the α phosphate group of ATP [68]. The present simulations show that this interaction is not formed upon the binding of Gαi and other ones are lost (for example between LYS 1065 and ATP, see Figure S8), when on the contrary, it is formed upon the binding of Gsα (see Figure 8). This structural effect of Gαi, which stabilizes the active site of AC5 in a state that prevents ATP to form the required interactions for catalysis, provides a structural basis for the enzymatic inhibition of AC5 by Gαi observed experimentally [24].

The binding of Gαi inhibits AC5 by increasing the flexibility of the active site allowing a high mobility of ATP without changing the complementarity of the C1/C2 interface. It was proposed previously [24] that the inhibitory effect of Gαi might be due to the disruption of the active site of AC5 or a decrease of the affinity between C1 and C2 domains. Our simulation study is in line with this first hypothesis. The further inactivation of AC5 + ATP by Gαi does not exclude the possibility that ATP is bound to AC5 during the inhibition process, and it may have a role in the tight regulation of the enzyme.

Based on our previous study [25] and the current one, we can speculate on the regulation mechanism of AC5 by considering only the Gαi_tilted configuration. In the absence of ATP, based on our simulations, only Gsα could interact with AC5, due to the close conformation of the binding site on the C1 domain. However, we cannot exclude the existence of Gαi + AC5 based on our study. The binding of ATP induces an opening of the angle between the pair of α-helices of domain C1 (α_C1_) and a closure of the pair of α-helices of domain C2 (α_C2_), which have a similar value close to the one observed upon the binding of Gsα. If Gαi binds first AC5 + ATP, the closure of the angle α_C2_ does not allow the binding of Gsα, and the ATP pocket remains close. When Gαi dissociates from AC5 + ATP, AC5 undergoes a conformational change that allows the binding of Gsα. On the contrary, if Gsα binds AC5 first, the enzyme is active thanks to the stabilization of ATP in its pocket and the formation of specific hydrogen bonds and cycles between AC5 + ATP + Gsα (favorable to Gαi binding) and AC5 + Gsα (unfavorable to Gαi binding). If Gsα dissociates, after cAMP release, AC5 is in an apo conformation, and there is no need for further inhibition. If Gsα dissociates from AC5 + ATP, then the conformation of AC5 becomes accessible to Gαi for the inhibition. Another possibility is that due to the open conformation of the binding site on the C1 domain, Gαi can bind to the AC5 + ATP + Gsα complex to form a ternary complex, whose existence is still unknown.

## 5. Conclusions

We perform all-atom molecular dynamics simulations in an attempt to better understand the regulation of adenylyl cyclase, which is a key enzymatic player in cellular signaling cascades. Microsecond-scale simulations of the G-protein subunit Gαi bound to the cytoplasmic domains of adenylyl cyclase in the presence and in the absence of ATP in two different conformations help to better understand some features of this important signal transmission protein, since no structural information on this complex is available. They notably provide information on a single, non-chimeric adenylyl cyclase isoform, AC5, which is bound to the inhibitory G-protein in the presence and in the absence of ATP.

The simulations show that protein binding creates significant changes in the structure and in flexibility throughout AC5 and due to a strong allosteric coupling existing within AC5 in a different fashion than the stimulatory G-protein and ATP. They provide data that help to explain the inhibition action of Gαi, whose binding increases the conformational and positional fluctuations of ATP in the active site of AC5 and its flexibility by moving away the key residues involved in the enzymatic reactions.

Our results also show that Gαi binding to the C1 domain does not impact C1/C2 interface complementarity, flexibilizes the C1 domain, and significantly closes the angle between the C2 α-helices that cannot bind Gsα when Gαi is in tilted conformation. The simultaneous binding of ATP and Gαi in a tilted conformation at the AC5 interface results in a rigidification of the C2 domain, without affecting the C1/C2 interface complementarity, and a slight increase of the angle between the C2 α-helices. Hence, Gαi has an important impact on AC5 dynamics, and its effects are enhanced when ATP binds by increasing the conformational freedom of the bound ligand, thus putting it in an unfavorable configuration within its binding site and not allowing the establishment of key interactions between ATP and AC5, which therefore leaves AC5 completely inactive.

Our simulations also show that ATP has a crucial role in the regulation of AC5, and we cannot exclude the presence of ATP during the inhibition. Our previous simulations already showed that ATP binding could influence the binding of the inhibitory G-protein subunit αi at the C1 domain. Here, we propose that the presence of ATP is needed to induce the competition between Gsα and Gαi to tightly regulate AC5. However, based on our results, we cannot exclude the existence of the Gαi + AC5 + ATP + Gsα complex and the Gαi + AC5 + Gsα complex. For the latter, other molecular dynamic studies of this hypothetical ternary complex concluded that if it exists, it would be inactive [29,30]. Further studies have to be conducted to shed light on this point.

## Supplementary Materials

The following are available online at https://www.mdpi.com/2218-273X/10/9/1330/s1, Figure S1: Gαi + AC5 + ATP complexes, viewed from the membrane side. Gαi is colored in purple, AC5 is colored in blue (C1 domain) and red (C2 domain). In green, residues labeled as “attractive” for the docking: residues 484–486 and 554–558 in AC5 (numbered according to P84309), which are at the entry of the binding site suggested by mutations in [24], and residues 209–216 in Gαi (numbered according to P08752), which are in the switch II region (201–221) involved in the binding [24]. The switch I and switch III regions [75] are indicated by arrows. Figure S2. Superimposition of the Gαi + AC5 + ATP complexes simulated in this study superimposed with the full-length structure of AC9 bound to Gsα (PDB structure 6R3Q [41]). Blue: C1 domain of AC5, red: C2 domain of AC5, purple: Gαi, gray: full-length AC9 bound with Gsα. Membrane location from [41]. Figure S3. Number of contacts at the AC5/Gαi interface. Contacts are defined using a 5 Å cut-off between heavy atoms. Top row: Fraction of initial contacts (t = 0) that are maintained as a function of time. Bottom row: total number of contacts between AC5 and Gαi; dashed horizontal lines indicate the number of contacts at t = 0. Figure S4: Snapshots of the Gαi + AC5 complexes observed during the simulations without ATP, viewed from the membrane side. Structures extracted every 250 ns are colored on a rainbow scale from blue to red. The C1 domain of AC5 is colored in gray, and the C2 domain is colored in beige. Figure S5: RMSD distribution for C1 and C2 domains, with respect to average structures, in the simulations with and without ATP. Data for AC5 and AC5 + Gsα, with and without ATP, taken from [25,72]. All the densities are computed with ggplot2 in R [66,67], using default parameters (Gaussian kernel density estimator and 512 equally spaced points). Figure S6: Substates of ATP and C2 observed in the Gαi_sym + AC5 + ATP simulation. A: Structural clusters obtained using gromos (cutoff = 1.5 Å on backbone atoms for C2 and cutoff = 1 Å for ATP), B: average structures viewed from the membrane side, C: close-up view on the β4 loop, from the cytoplasmic side. The average structures are colored in gray (400 ns < T < 1100 ns) and yellow (1200 ns < T < 1500 ns). Figure S7: RMSD distribution for ATP. Data for AC5 + ATP and AC5 + ATP + Gsα taken from [25,39]. Figure S8: Distance between ATP and key residues of C2. Data for AC5 + ATP and AC5 + ATP + Gsα are from our previous study [25,72]. ATP/ARG1029: distance between the O2α of ATP and the center of mass of terminal hydrogen atoms, which are covalently bound to Nε of ARG 1029. ATP/LYS1065: distance between the O2γ of ATP and the center of mass of the terminal hydrogen atoms, which are covalently bound to Nζ of LYS 1065. Figure S9: Distribution of the angles between helix axes (helix α1 and helix α2 in C1, helix α3 and helix α4 in C2, see Figure 3). Data for AC5 and AC5 + Gsα, with and without ATP, taken from [25,72]. Figure S10: Distribution of the distance between helix axes (helix α1 and helix α2 in C1, helix α3 and helix α4 in C2, see Figure 3). Data for AC5 and AC5 + Gsα, with and without ATP, taken from [25,39]. Figure S11: Distribution of the C1/C2 interface gap index. Data for AC5 and AC5 + Gsα, with and without ATP, taken from [25,72]. Figure S12. Local comparison of C2 loops in average structures without ATP. White: AC5, red/blue: Gαi_tilted + AC5, pink/cyan: Gαi_sym + AC5. Left panel: β2 loop, right panel: β4 loop. Figure S13. Substates of domain C2 observed during the simulation of Gαi_sym + AC5 complex, without ATP. Left: RMSD time series for the C2 domain, colored according to cluster membership. Right: Structures closest to the center of each cluster, and the relative size of each cluster as percentages. Prominent structural changes are indicated by red arrows. Figure S14. The persistent interface residues in Gαi_tilted + AC5 + ATP simulation. The C1 domain is colored in blue, the C2 domain is colored in red, Gαi is colored in purple. Important residues on C1 from reference [24] that are persistent at the interface with Gαi are shown as sticks, which are colored in orange and labeled with a residue numbering from [24]. Residues on Gαi that form stable interactions from Table S5 are shown in sticks and colored in pink (switch I), yellow (switch II), and cyan (switch III). Table S1. Average and standard deviation of backbone RMSD for the C1 and C2 domains of AC5, angle between helices (αC1 and αC2), distance between helices axes (dC1 and dC2), Gap index for the interface C1/C2, RMSD for ATP. a: data in italics are from our previous study [25,39]. Table S2: Mean values and standard deviation of Gap index for the Gαi/AC5 interface. a: data in italics are from our previous study for the Gap index for the Gsα/AC5 interface [25,72]. Table S3. Distance between Mg^2+^ ions and the aspartate residues (ASP 396 and ASP 440 in C1 domain). Table S4. Persistence of residues of AC5 mutated in reference [24] at AC5/Gαi interface in our simulations. The fraction of time each residue is part of the AC5/Gαi interface is shown as a percentage, and the number of contacting residues on Gαi is between parentheses. Interface residues are defined using a 5 Å cut-off between heavy atoms. Residues in italics have limited or no impact on the binding. ^1^: For the sake of comparison, residue numbering refers to reference [24]; the corresponding numbers in Uniprot sequence P84309 are between parentheses. ^2^: In Ref. [24], the double mutant M414/T415 was studied; hence, we monitor the presence of M414 or T415. Table S5. Stable interactions between key residues of AC5 from reference [24] and residues of Gαi. Interactions that appear more than 50% of the time in each simulation are listed with the corresponding fraction of time between parentheses. Interactions are defined using a 5 Å cut-off between heavy atoms. Interactions that are common to simulations with and without ATP for a given system are highlighted in bold. For the sake of comparison, AC5 residue numbering refers to reference [24]; the corresponding numbers in Uniprot sequence P84309 are between parentheses. Gαi numbering refers to the Uniprot sequence. ^1^: Gαi residues belonging to the switch I region, ^2^: Gαi residues belonging to the switch II region, ^3^: Gαi residues belonging to the switch III region [75].
