# Supplementary material for: Allosteric Inhibition of Adenylyl Cyclase Type 5 by G-Protein: A Molecular Dynamics Study
Source: Biomolecules. 2020 Sep 17;10(9):1330. doi: 10.3390/biom10091330 (PMC7563791; doi:10.3390/biom10091330)
Supplement: Supplementary file 1 [file biomolecules-10-01330-s001.pdf]

SUPPLEMENTARY MATERIALS

Allosteric inhibition of adenylyl cyclase type 5 by G-protein: a molecular dynamics study

Elisa Frezza[1*], Tina-Méryl Amans[2], Juliette Martin[2*]

[1]Université de Paris, CiTCoM, CNRS, F-75006 Paris, France
[2]University of Lyon, CNRS, UMR5086 Molecular Microbiology and Structural Biochemistry, Lyon, France

Corresponding authors: * elisa.frezza@u-paris.fr (EF), juliette.martin@ibcp.fr (JM)

S1. Figures

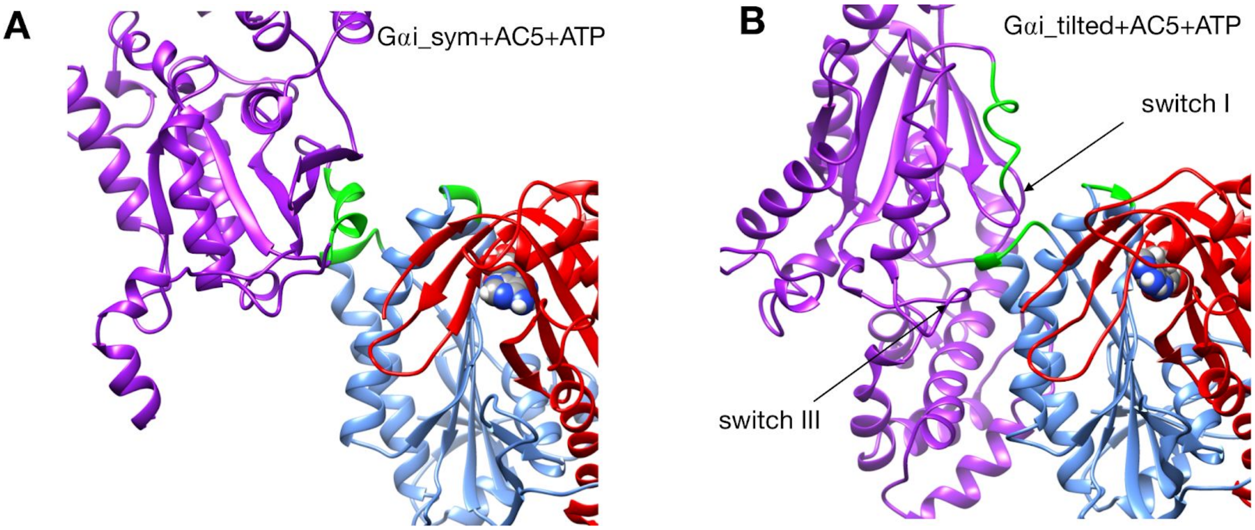

Figure S1: Gαi+AC5+ATP complexes, viewed from the membrane side. Gαi is colored in purple, AC5 in blue (C1 domain) and red (C2 domain). In green, residues labeled as "attractive" for the docking: residues 484-486 and 554-558 in AC5 (numbered according to P84309), which are at the entry of the binding site suggested by mutations in [24], and residues 209-216 in Gαi (numbered according to P08752), which are in the switch II region (201-221) involved in the binding [24]. The switch I and switch III regions [75] are indicated by arrows.

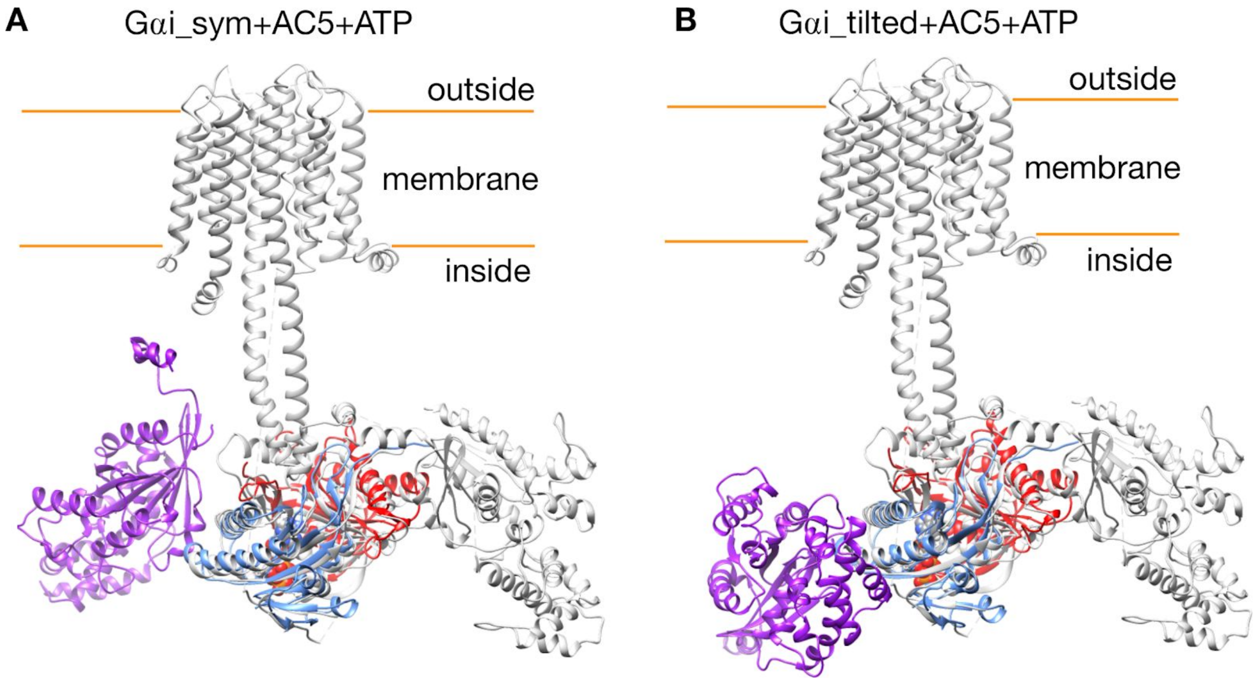

Figure S2. Superimposition of the Gαi+AC5+ATP complexes simulated in this study superimposed with the full-length structure of AC9 bound to Gsα (PDB structure 6R3Q [41]). Blue: C1 domain of AC5, red: C2 domain of AC5, purple: Gαi, grey: full-length AC9 bound with Gsα. Membrane location from [41].

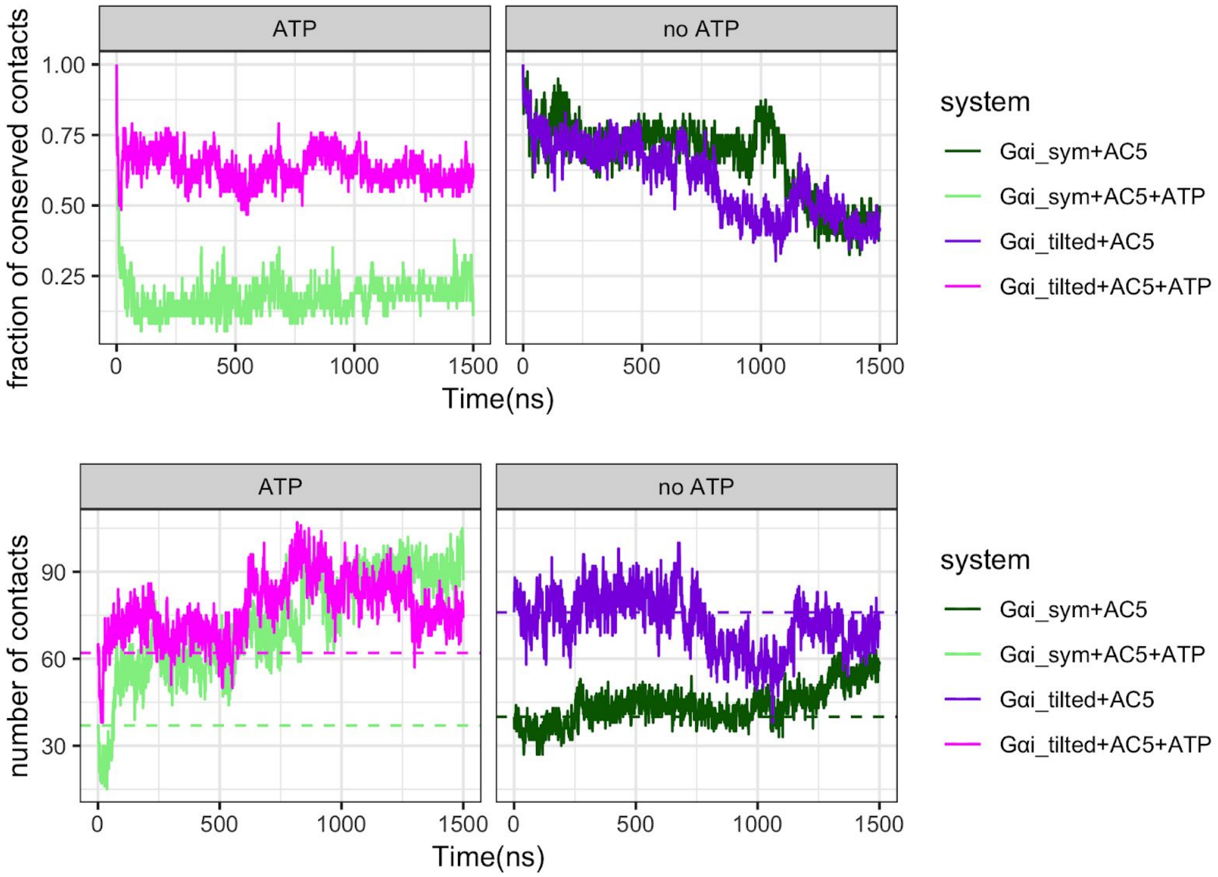

Figure S3. Number of contacts at the AC5/Gαi interface. Contacts are defined using a 5 Å cut-off between heavy atoms. Top row: fraction of initial contacts (t=0) that are maintained as a function of time. Bottom row: total number of contacts between AC5 and Gαi; dashed horizontal lines indicate the number of contacts at t=0.

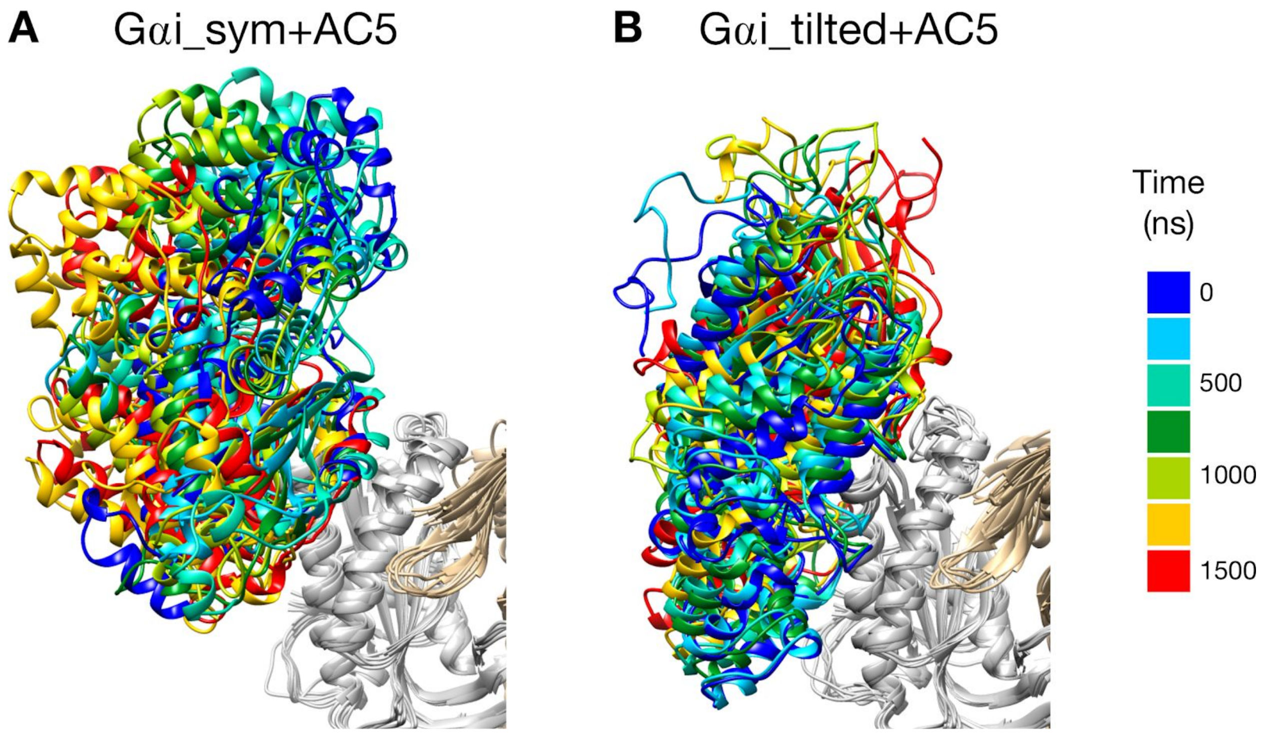

Figure S4. Snapshots of the Gαi+AC5 complexes observed during the simulations without ATP, viewed from the membrane side. Structures extracted every 250 ns are colored on a rainbow scale from blue to red. The C1 domain of AC5 is colored in grey and the C2 domain in beige.

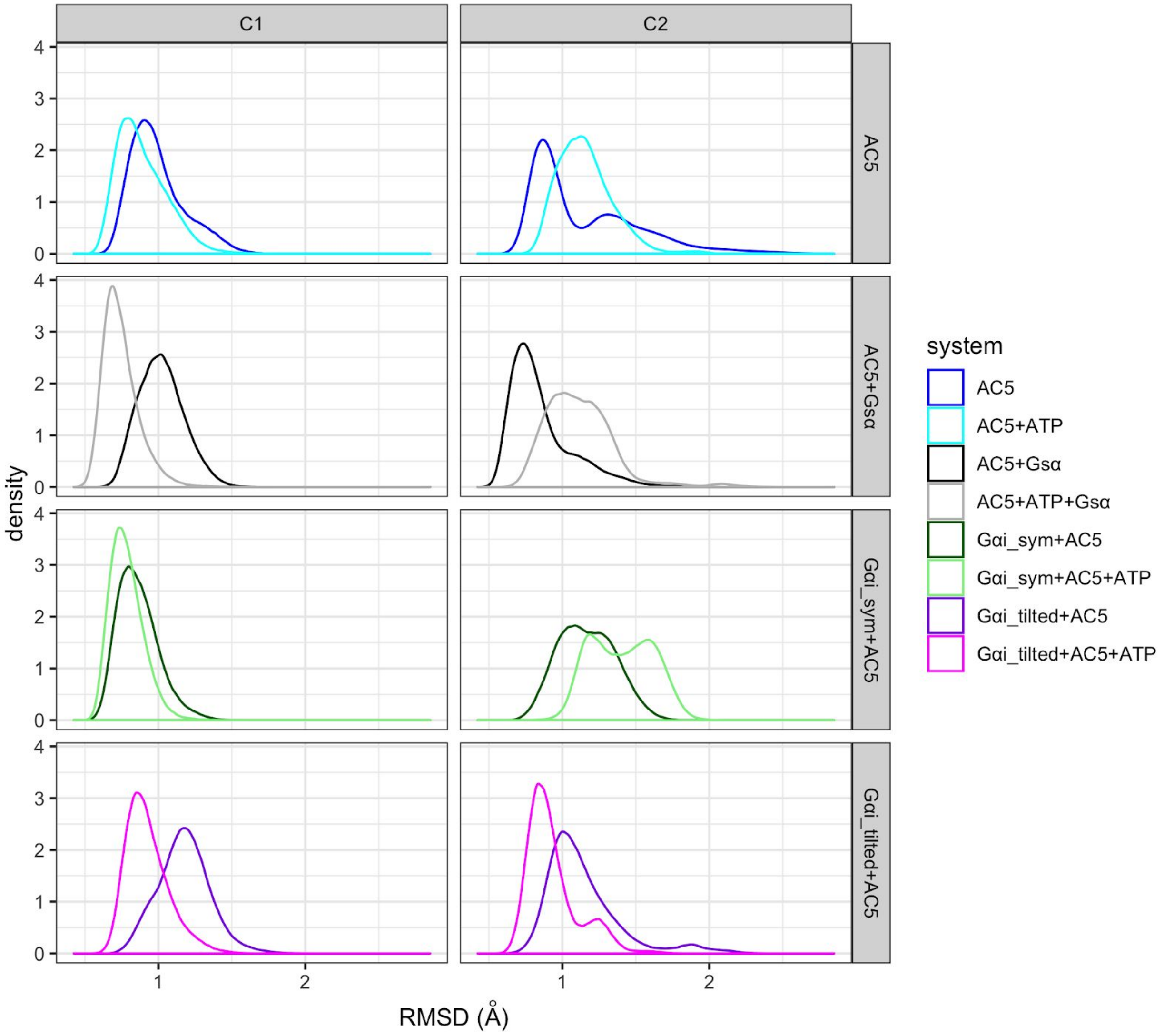

Figure S5: RMSD distribution for C1 and C2 domains, with respect to average structures, in the simulations with and without ATP. Data for AC5 and AC5+Gsα, with and without ATP, taken from [25,71]. Densities are computed with ggplot2 in R [66,67], using default parameters (Gaussian kernel density estimator and 512 equally spaced points).

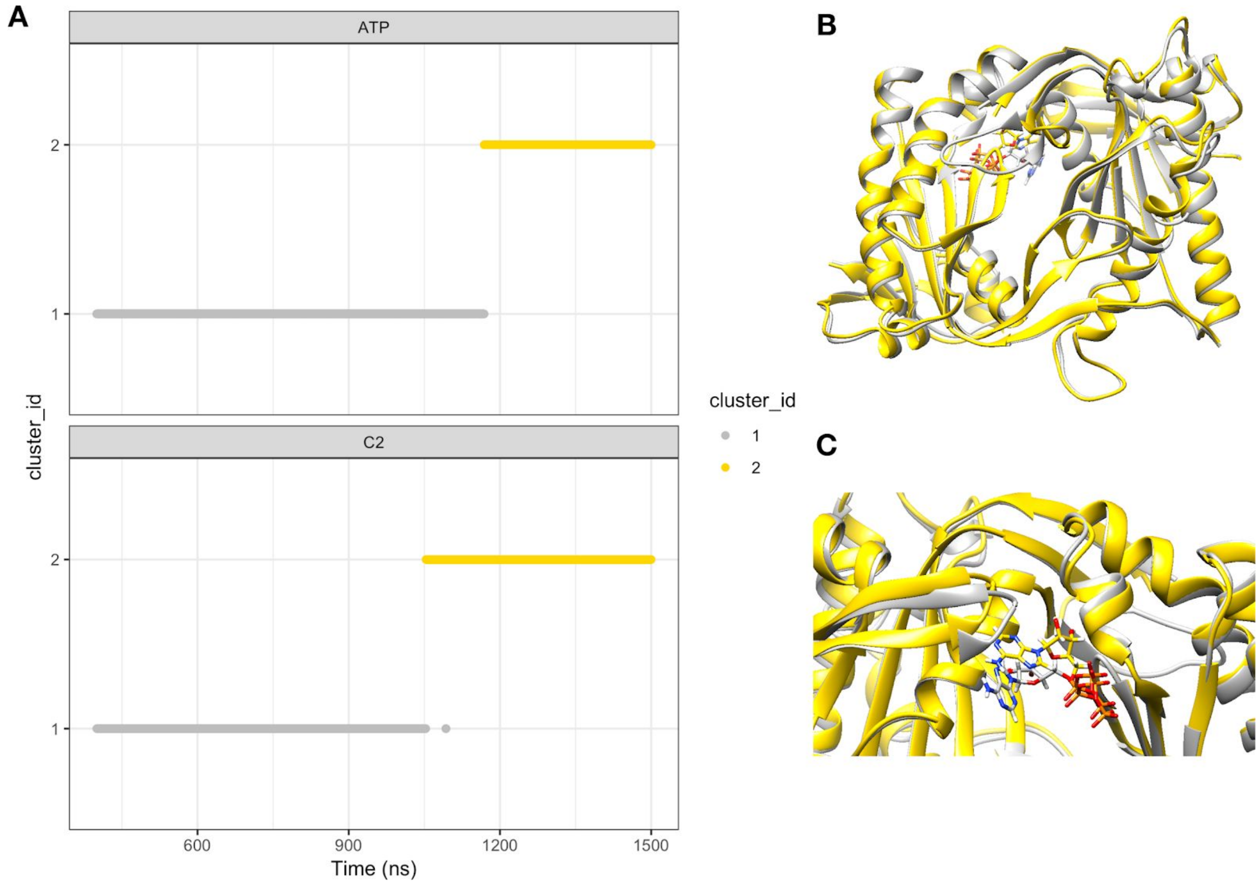

Figure S6: Sub-states of ATP and C2 observed in the Gαi_sym+AC5+ATP simulation. A: structural clusters obtained using gromos (cutoff=1.5 Å on backbone atoms for C2 and cutoff=1 Å for ATP), B: average structures viewed from the membrane side, C: close-up view on the β4 loop, from the cytoplasmic side. The average structures are colored in grey (400ns<t<1100 ns) and yellow (1200ns<t<1500ns).

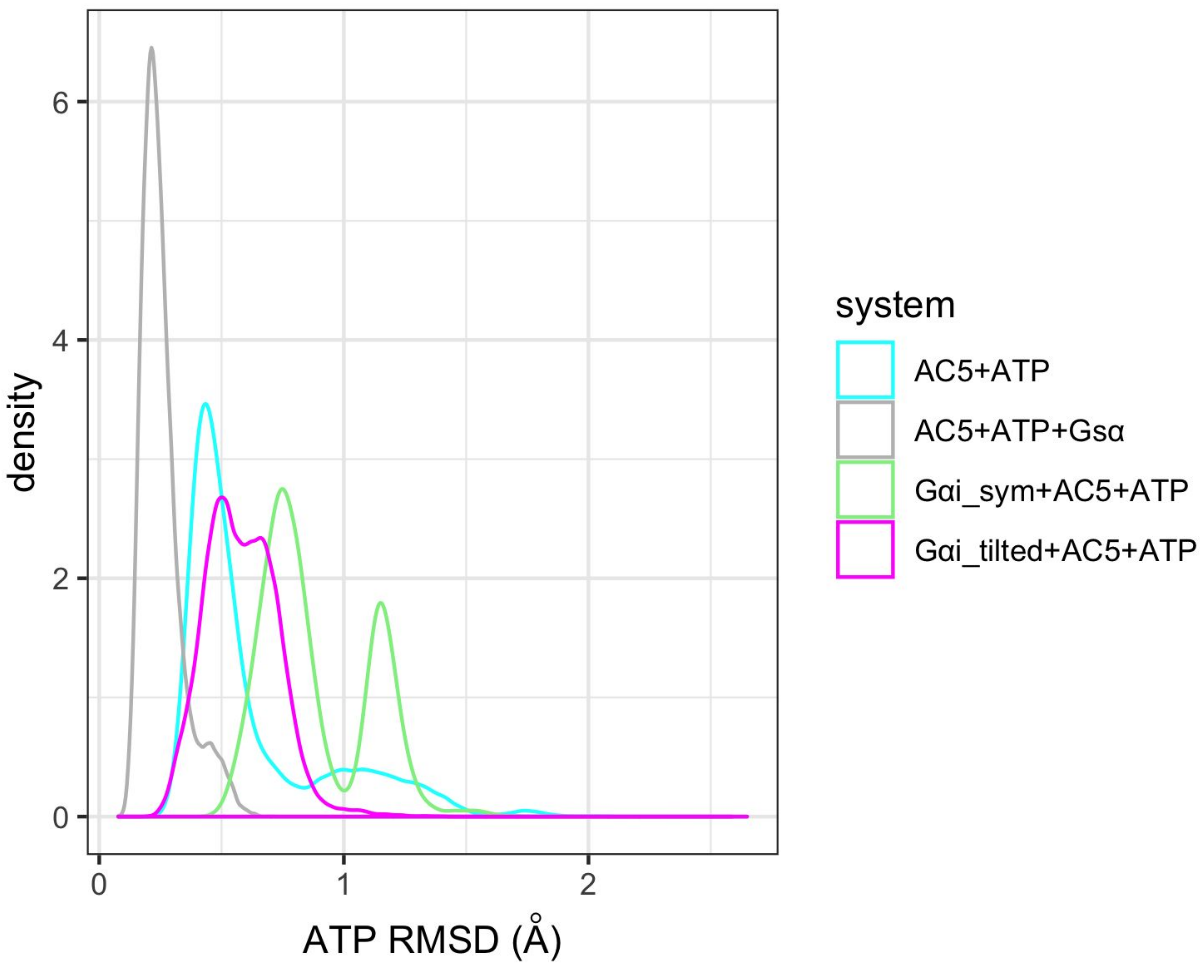

Figure S7: RMSD distribution for ATP. Data for AC5+ATP and AC5+ATP+Gs α taken from [25,71].

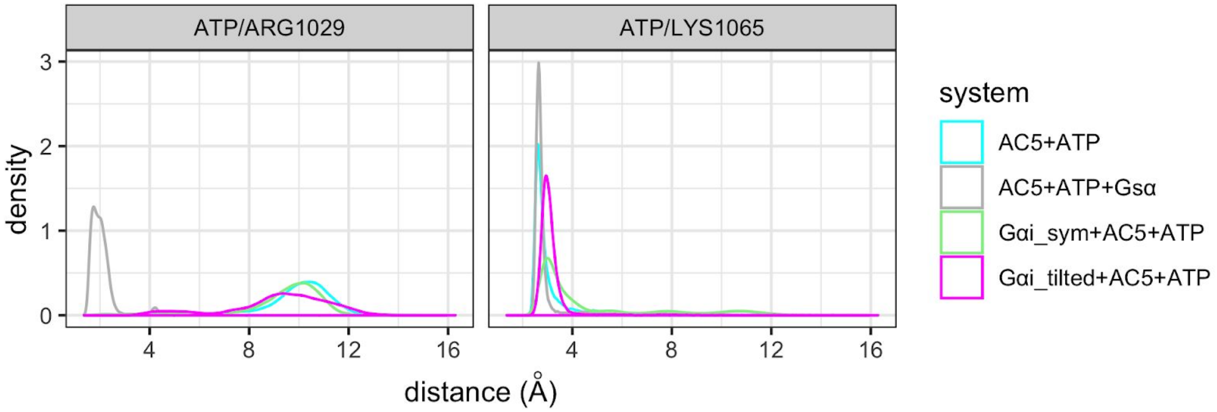

Figure S8: Distance between ATP and key residues of C2. Data for AC5+ATP and AC5+ATP+Gs α are from our previous study [25,71]. ATP/ARG1029: distance between the O2 α of ATP and the center of mass of terminal hydrogen atoms which are covalently bound to N ε of ARG 1029. ATP/LYS1065: distance between the O2 γ of ATP and the center of mass of the terminal hydrogen atoms which are covalently bound to N ζ of LYS 1065.

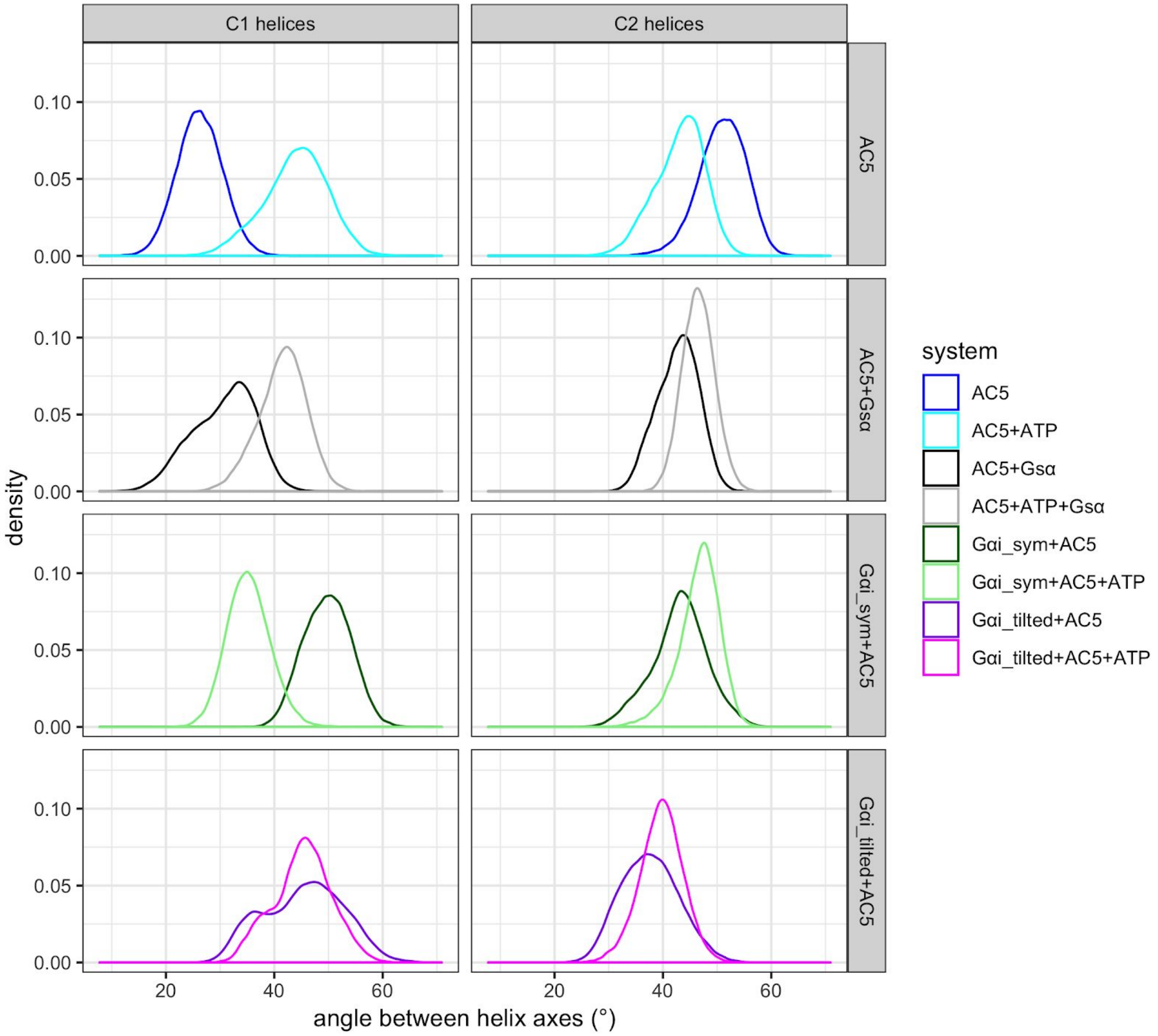

Figure S9: Distribution of the angles between helix axes (helix $\alpha 1$ and helix $\alpha 2$ in C1, helix $\alpha 3$ and helix $\alpha 4$ in C2, see Figure 3). Data for AC5 and AC5+Gsα, with and without ATP, taken from [25,71].

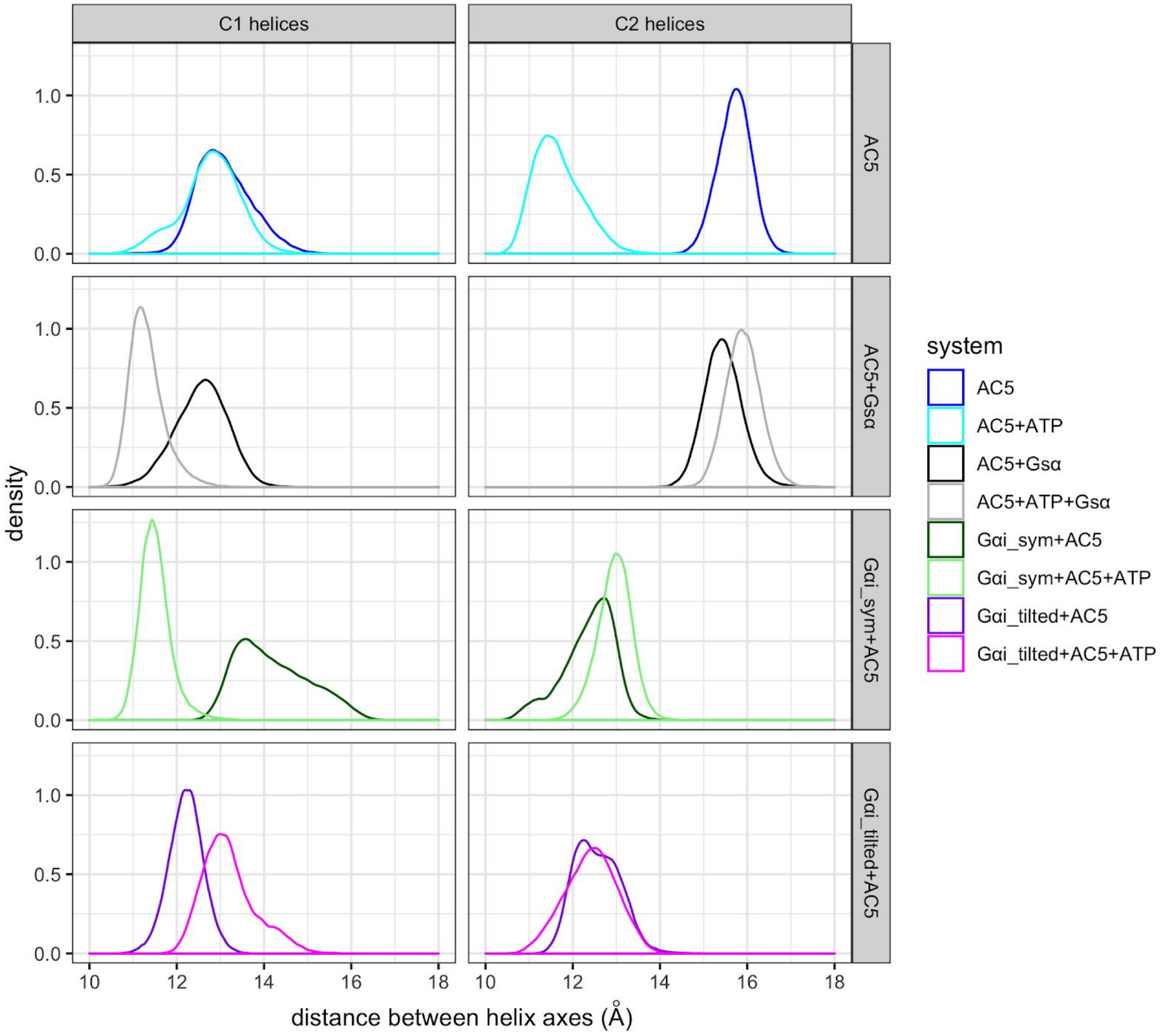

Figure S10: Distribution of the distance between helix axes (helix $\alpha 1$ and helix $\alpha 2$ in C1, helix $\alpha 3$ and helix $\alpha 4$ in C2, see Figure 3). Data for AC5 and AC5+Gsα, with and without ATP, taken from [25,71].

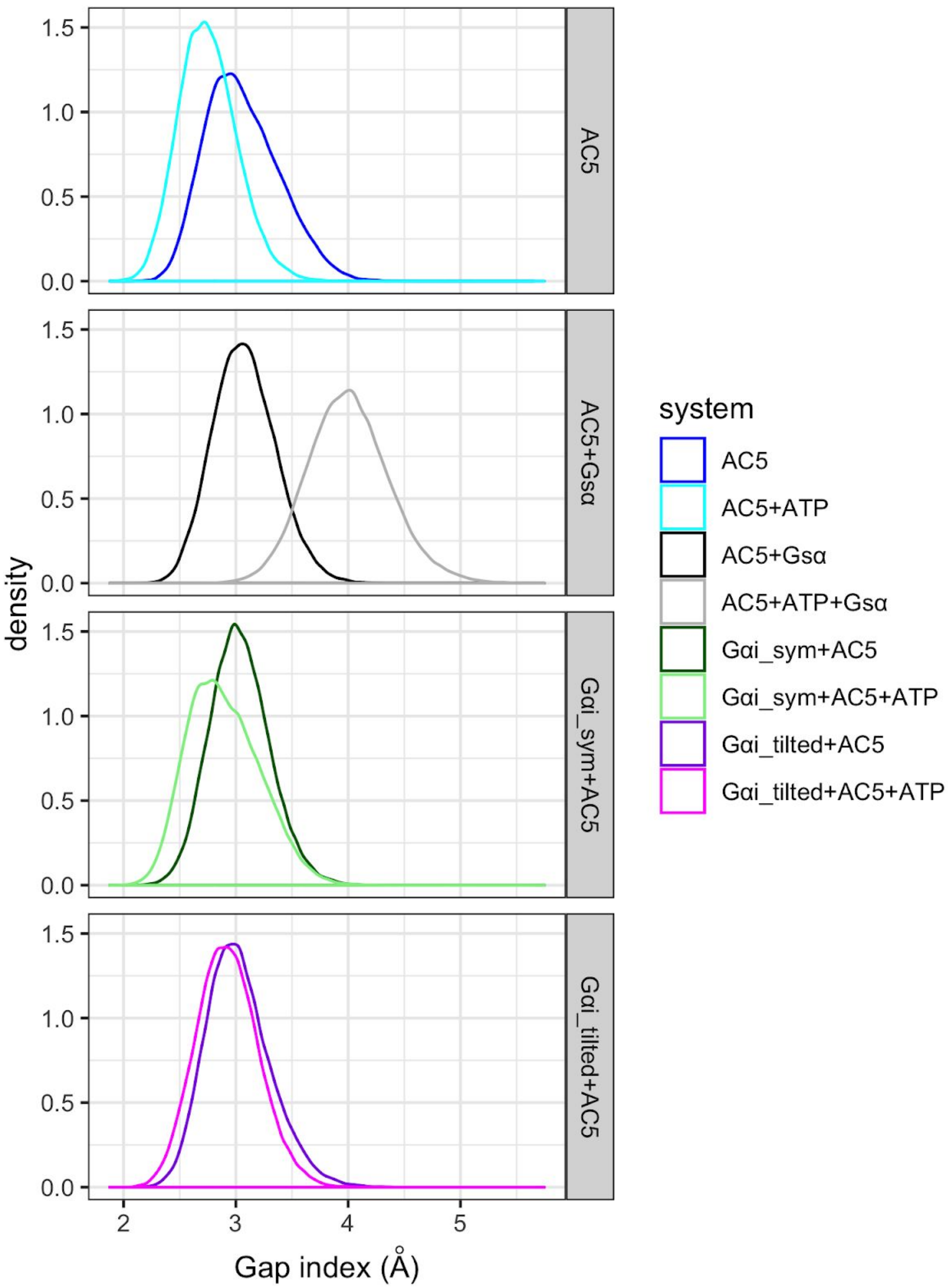

Figure S11: Distribution of the C1/C2 interface Gap index. Data for AC5 and AC5+Gsα, with and without ATP, taken from [25,71].

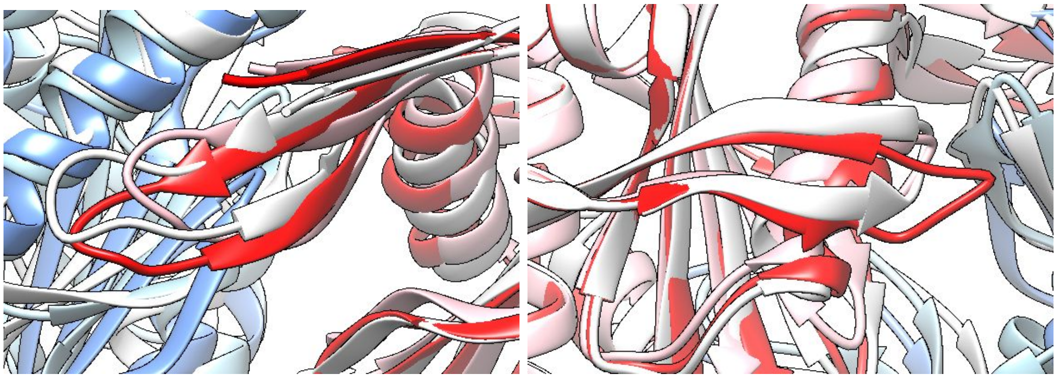

Figure S12. Local comparison of C2 loops in average structures without ATP. White: AC5, red/blue: Gαi_tilted+AC5, pink/cyan: Gαi_sym+AC5. Left panel: β2 loop, right panel: β4 loop.

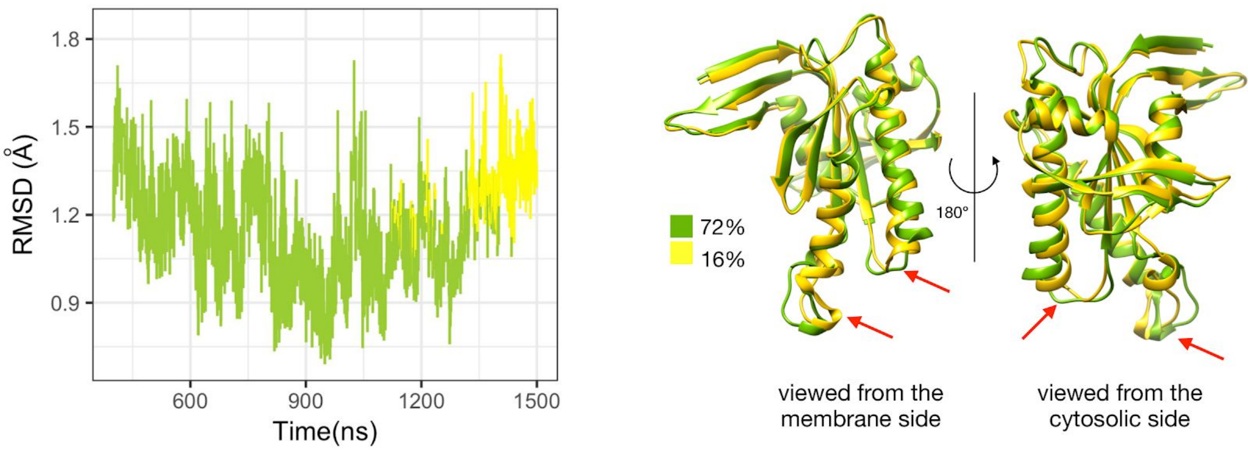

Figure S13. Sub-states of domain C2 observed during the simulation of Gαi_sym+AC5 complex, without ATP. Left: RMSD time series for the C2 domain, colored according to cluster membership. Right: structures closest to the center of each cluster, and relative size of each cluster as percentages. Prominent structural changes are indicated by red arrows.

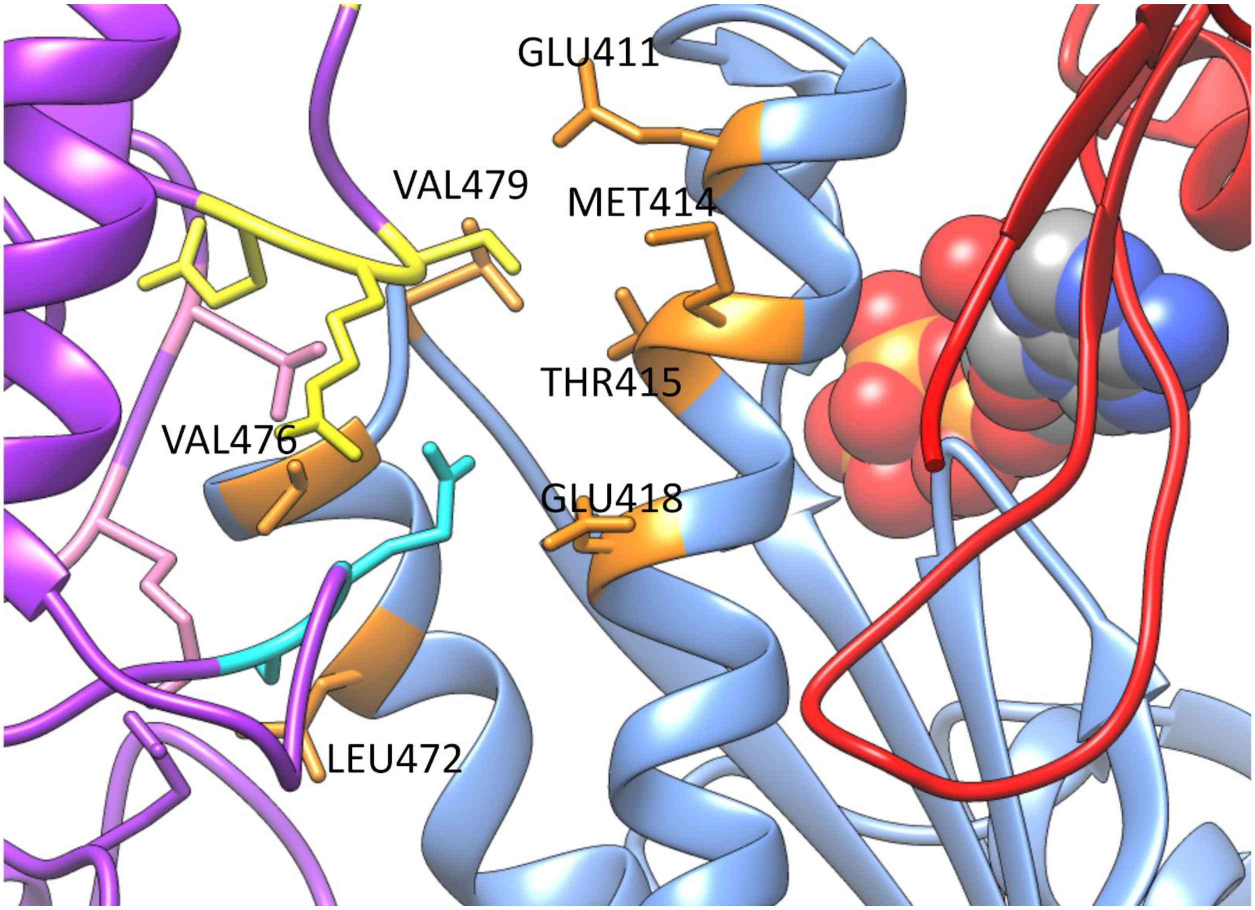

Figure S14. The persistent interface residues in Gαi_tilted+AC5+ATP simulation. The C1 domain is colored in blue, the C2 domain in red, Gαi in purple. Important residues on C1 from reference [24] that are persistent at the interface with Gαi are shown as sticks, colored in orange and labeled with residue numbering from [24]. Residues on Gαi that form stable interactions from Table S5 are shown in sticks and colored in pink (switch I), yellow (switch II) and cyan (switch III).

S2. Tables

Table S1. Average and standard deviation of backbone RMSD for the C1 and C2 domains of AC5, angle between helices (α_{C1} and α_{C2}), distance between helices axes (d_{C1} and d_{C2}), Gap index for the interface C1/C2, RMSD for ATP. [a]: data in italic are from our previous study [25,71].

System	RMSD C1 (Å)	RMSD C2 (Å)	α_{C1} (°)	d_{C1} (Å)	α_{C2} (°)	d_{C2} (Å)	Gap index C1/C2 (Å)	RMSD ATP (Å)
AC5+ATP[a]	*0.9 ± 0.2*	*1.2 ± 0.2*	*44 ± 6*	*12.8 ± 0.7*	*43 ± 5*	*11.7 ± 0.6*	*2.8 ± 0.3*	*0.6 ± 0.3*
AC5+ATP+Gsα[a]	*0.8 ± 0.1*	*1.1 ± 0.2*	*42 ± 5*	*11.3 ± 0.4*	*47 ± 3*	*15.9 ± 0.4*	*3.8 ± 0.5*	*0.3 ± 0.1*
Gαi_sym+AC5+ATP	0.8 ± 0.1	1.4 ± 0.2	35 ± 4	11.5 ± 0.5	47 ± 4	13.0 ± 0.4	2.9 ± 0.3	0.9 ± 0.2
Gαi_tilted+AC5+ATP	0.9 ± 0.1	0.9 ± 0.2	45 ± 5	13.2 ± 0.6	40 ± 4	12.4 ± 0.6	2.9 ± 0.3	0.6 ± 0.1
AC5[a]	*1.0 ± 0.2*	*1.2 ± 0.4*	*26 ± 4*	*13.1 ± 0.6*	*50 ± 4*	*15.7 ± 0.4*	*3.1 ± 0.3*	
AC5+Gsα[a]	*1.0 ± 0.2*	*0.9 ± 0.2*	*31 ± 6*	*12.6 ± 0.6*	*43 ± 4*	*15.4 ± 0.4*	*3.1 ± 0.3*	
Gαi_sym+AC5	0.9 ± 0.1	1.2 ± 0.2	50 ± 4	14.2 ± 0.8	43 ± 5	12.4 ± 0.6	3.0 ± 0.3	
Gαi_tilted+AC5	1.2 ± 0.2	1.1 ± 0.3	45 ± 7	12.2 ±0.4	38 ± 5	12.6 ± 0.5	3.0 ± 0.3	

Table S2: Mean values and standard deviation of Gap index for the Gαi/AC5 interface. [a]: data in italic are from our previous study for the Gap index for the Gsα/AC5 interface [25,71].

System	Gap Volume (Å^3)	ΔASA (Å^2)	Gap index (Å)
Gαi_sym+AC5+ATP	7569 ± 504	1413 ± 157	5.4 ± 0.7
Gαi_tilted+AC5+ATP	5640 ± 469	1345 ± 152	4.2 ± 0.6
Gαi_sym+AC5	2850 ± 449	881 ± 101	3.2 ± 0.4
Gαi_tilted+AC5	5561 ± 562	1225 ± 138	4.6 ± 0.7
AC5+ATP+Gsα[a]	*3286 ± 333*	*1244 ± 123*	*2.7 ± 0.5*
AC5+Gsα[a]	*3424 ± 361*	*1067 ± 63*	*3.2 ± 0.4*

Table S3. Distance between Mg^{2+} ions (MG1 and MG2) and the aspartate residues (ASP 396 and ASP 440 in C1 domain).

**System**	MG1/ASP396 (Å)	MG1/ASP440 (Å)	MG2/ASP396 (Å)	MG2/ASP440 (Å)
AC5+ATP[a]	2.5 ± 0.1	2.4 ± 0.1	2.6 ± 0.1	2.6 ± 0.1
AC5+ATP+Gsα[a]	2.4 ± 0.1	2.4 ± 0.1	2.6 ± 0.1	2.6 ± 0.1
Gαi_sym+AC5+ATP	2.9 ± 0.1	7.2 ± 0.9	2.6 ± 0.1	5.2 ± 0.3
Gαi_tilted+AC5+ATP	2.5 ± 0.1	2.4± 0.1	2.6 ± 0.1	2.6 ± 0.1

Table S4. Persistence of residues of AC5 mutated in reference [24] at AC5/Gαi interface in our simulations. The fraction of time each residue is part of the AC5/Gαi interface is shown as percentage, and the number of contacting residues on Gαi is between parentheses. Interface residues are defined using a 5 Å cut-off between heavy atoms. Residues in italic have limited or no impact on the binding. [1]: for the sake of comparison, residue numbering refers to reference [24]; the corresponding numbers in Uniprot sequence P84309 are between parentheses. [2]: in ref[24], the double mutant M414/T415 was studied, hence we monitor the presence of M414 or T415.

Residue number [1]	Gαi_sym+AC5+ATP	Gαi_tilted+AC5+ATP	Gαi_sym+AC5	Gαi_tilted+AC5
GLU398 (477)	*0 % (0.0)*	*0 % (0.0)*	*0 % (0.0)*	*0 % (0.0)*
SER402 (481)	*0 % (0.0)*	*6 % (0.1)*	*0 % (0.0)*	*10 % (0.1)*
GLN406 (485)	*50 % (0.7)*	*55 % (1.3)*	*85 % (1.1)*	*90 % (1.3)*
GLU411 (490)	100 % (4.4)	81 % (2.1)	98 % (2.0)	99 % (2.1)
MET414(493)	100 % (3.6)	60 % (1.1)	100 % (2.4)	64 % (1.1)
THR415(494)	28 % (0.3)	76 % (0.8)	96 % (1.7)	98 % (0.8)
MET414 or T415[2]	100%	84%	100%	99%
GLU418 (497)	10 % (0.1)	96 % (1.8)	100 % (3.4)	92 % (1.8)
LEU472 (551)	0 % (0.0)	98 % (2.1)	100 % (3.8)	96 % (2.1)
VAL476 (555)	97 % (1.3)	100 % (4.7)	100 % (6.9)	100 % (4.7)
VAL479 (558)	3 % (0.0)	99 % (3.2)	96 % (1.6)	100 % (3.2)
ASN480 (559)	*0 % (0.0)*	*97 % (2.7)*	*5 % (0.0)*	*85 % (2.7)*
ALA523 (602)	*0 % (0.0)*	*65 % (0.9)*	*0 % (0.0)*	*33 % (0.9)*

Table S5. Stable interactions between key residues of AC5 from reference [24] and residues on Gαi. Interactions that appear more than 50% of the time in each simulation are listed with the corresponding fraction of time between parentheses. Interactions are defined using a 5 Å cut-off between heavy atoms. Interactions that are common to simulations with and without ATP for a given system are highlighted in bold. For the sake of comparison, AC5 residue numbering refers to reference [24]; the corresponding numbers in Uniprot sequence P84309 are between parentheses. Gαi numbering refers to the Uniprot sequence. ^{1}: Gαi residues belonging to the switch I region, ^{2}: Gαi residues belonging to the switch II region, ^{3}: Gαi residues belonging to the switch III region [75].

AC5 residue	Gαi_sym+AC5+ATP	Gαi_tilted+AC5+ATP	Gαi_sym+AC5	Gαi_tilted+AC5
GLU411 (490)	THR183 (65%)^{1}<br>GLY184 (92%)^{1}<br>ILE185 (98%)^{1}<br>**LYS211 (99%)**^{2}	**SER207 (57%)**^{2}<br>**LYS210 (64%)**^{2}	LYS210 (67%)^{2}<br>**LYS211 (52%)**^{2}	**SER207 (96%)**^{2}<br>**LYS210 (61%)**^{2}
MET414(493)	ILE185 (55%)^{1}<br>CYS215 (91%)^{2}<br>**HIS214 (96%)**^{2}	-	ILE213 (52%)^{2}<br>**HIS214 (93%)**^{2}	SER207 (59%)^{2}
THR415(494)	-	**SER207 (76%)**^{2}	ILE213 (81%)^{2}	**SER207 (98%)**^{2}
GLU418 (497)	-	**ARG206 (90%)**^{2}<br>**GLU237 (85%)**^{3}	ILE213 (99%)^{2}<br>HIS214 (60%)^{2}<br>GLU217 (56%)^{2}<br>TRP259 (87%)	**ARG206 (80%)**^{2}<br>**GLU237 (86%)**
LEU472 (551)	-	**GLN148 (79%)**<br>ALA236 (67%)^{3}	N257 (100%)<br>LYS258 (73%)<br>TRP259 (99%)	**GLN148 (75%)**
VAL476 (555)	HIS214 (95%)^{2}	**GLN205 (99%)**^{2}<br>**ARG206 (97%)**^{2}<br>**ALA236 (74%)**^{3}<br>**GLU237 (86%)**^{3}	ARG209 (71%)^{2}<br>TRP212 (100%)^{2}<br>ILE213 (89%)^{2}<br>SER253 (67%)<br>ILE254 (94%)<br>ASN257(74%)<br>TRP259 (70%)<br>PHE260 (64%)	**GLN205 (90%)**^{2}<br>**ARG206 (100%)**^{2}<br>SER207 (59%)^{2}<br>LEU235 (63%)^{3}<br>**ALA236 (57%)**^{3}<br>**GLU237 (81%)**^{3}
VAL479 (558)	-	LYS181 (56%)^{1}<br>THR183 (73%)^{1}<br>**SER207 (93%)**^{2}	ARG206 (56%)^{2}<br>LYS210 (67%)^{2}	**SER207 (100%)**^{2}<br>GLU208 (95%)^{2}
